# Nutrient Patterns and Their Food Sources in an International Study Setting: Report from the EPIC Study

**DOI:** 10.1371/journal.pone.0098647

**Published:** 2014-06-05

**Authors:** Aurelie Moskal, Pedro T. Pisa, Pietro Ferrari, Graham Byrnes, Heinz Freisling, Marie-Christine Boutron-Ruault, Claire Cadeau, Laura Nailler, Andrea Wendt, Tilman Kühn, Heiner Boeing, Brian Buijsse, Anne Tjønneland, Jytte Halkjær, Christina C. Dahm, Stephanie E. Chiuve, Jose R. Quirós, Genevieve Buckland, Esther Molina-Montes, Pilar Amiano, José M. Huerta Castaño, Aurelio Barricarte Gurrea, Kay-Tee Khaw, Marleen A. Lentjes, Timothy J. Key, Dora Romaguera, Anne-Claire Vergnaud, Antonia Trichopoulou, Christina Bamia, Philippos Orfanos, Domenico Palli, Valeria Pala, Rosario Tumino, Carlotta Sacerdote, Maria Santucci de Magistris, H. Bas Bueno-de-Mesquita, Marga C. Ocké, Joline W. J. Beulens, Ulrika Ericson, Isabel Drake, Lena M. Nilsson, Anna Winkvist, Elisabete Weiderpass, Anette Hjartåker, Elio Riboli, Nadia Slimani

**Affiliations:** 1 Dietary Exposure Assessment Group, International Agency for Research on Cancer, Lyon, France; 2 Nutritional Epidemiology Group, International Agency for Research on Cancer, Lyon, France; 3 Biostatistics Group, International Agency for Research on Cancer, Lyon, France; 4 Center for research in Epidemiology and Population Health, Nutrition, Hormones and Women’s Health, INSERM U1018, Villejuif, France; 5 Université Paris Sud, UMRS 1018, Villejuif, France; 6 Institut Gustave-Roussy, Villejuif, France; 7 Division of Cancer Epidemiology, German Cancer Research Center, Heidelberg, Germany; 8 Department of Epidemiology, German Institute of Human Nutrition, Potsdam-Rehbruecke, Germany; 9 Danish Cancer Society Research Center, Diet, Genes and Environment, Copenhagen, Denmark; 10 Section for Epidemiology, Institute of Public Health, Aarhus University, Aarhus, Denmark; 11 Division of Preventive Medicine, Department of Medicine, Brigham and Women’s Hospital and Harvard Medical School, Boston, Massachusetts, United States of America; 12 Department of Nutrition, Harvard School of Public Health, Boston, Massachusetts, United States of America; 13 Public Health and Health Planning Directorate, Asturias, Spain; 14 Unit of Nutrition, Environment and Cancer, Cancer Epidemiology Research Programme, Catalan Institute of Oncology, Barcelona, Spain; 15 Andalusian School of Public Health, Granada, Spain; 16 Consortium for Biomedical Research in Epidemiology and Public Health (CIBER Epidemiología y Salud Pública-CIBERESP), Madrid, Spain; 17 Public Health Division of Gipuzkoa, BIODonostia Research Institute, San Sebastian, Spain; 18 Navarre Public Health Institute, Pamplona, Spain; 19 Department of Epidemiology, Murcia Regional Health Council, Murcia, Spain; 20 Department of Public Health and Primary Care, University of Cambridge, Cambridge, United Kingdom; 21 Cancer Epidemiology Unit, University of Oxford, Oxford, United Kingdom; 22 Department of Epidemiology and Biostatistics, School of Public Health, Imperial College London, London, United Kingdom; 23 CIBER Fisiopatología de la Obesidad y Nutrición, Madrid, Spain; 24 WHO Collaborating Center for Food and Nutrition Policies, Department of Hygiene, Epidemiology and Medical Statistics, University of Athens Medical School, Athens, Greece; 25 Hellenic Health Foundation, Athens, Greece; 26 Molecular and Nutritional Epidemiology Unit, Cancer Research and Prevention Institute ISPO, Florence, Italy; 27 Nutritional Epidemiology Unit, Fondazione IRCCS Istituto Nazionale dei Tumori, Milan, Italy; 28 Cancer Registry and Histopathology Unit, “Civile - M.P. Arezzo” Hospital, Ragusa, Italy; 29 HuGeF Foundation and Center for Cancer Prevention CPO-Piemonte, Torino, Italy; 30 Department of Clinical and Experimental Medicine, Federico II University, Naples, Italy; 31 The National Institute for Public Health and the Environment (RIVM), Bilthoven, The Netherlands; 32 Department of Gastroenterology and Hepatology, University Medical Center, Utrecht, The Netherlands; 33 Julius Center for Health Sciences and Primary Care, University Medical Center Utrecht, Utrecht, The Netherlands; 34 Diabetes and Cardiovascular Disease, Genetic Epidemiology, Department of Clinical Sciences in Malmö, Lund University, Malmö, Sweden; 35 Department of Clinical Sciences in Malmö, Research Group in Nutritional Epidemiology, Lund University, Malmö, Sweden; 36 Department of Public Health and Clinical Medicine, Nutrition Research, Umeå University, Umeå, Sweden; 37 Department of Internal Medicine and Clinical Nutrition, University of Gothenburg, Gothenburg, Sweden; 38 Department of Community Medicine, Faculty of Health Sciences, University of Tromsø, Tromsø, Norway; 39 Department of Research, Cancer Registry of Norway, Oslo, Norway; 40 Department of Medical Epidemiology and Biostatistics, Karolinska Institutet, Stockholm, Sweden; 41 Samfundet Folkhälsan, Helsinki, Finland; 42 Department of Nutrition, Institute of Basic Medical Sciences, University of Oslo, Oslo, Norway; University College Dublin, Ireland

## Abstract

**Background:**

Compared to food patterns, nutrient patterns have been rarely used particularly at international level. We studied, in the context of a multi-center study with heterogeneous data, the methodological challenges regarding pattern analyses.

**Methodology/Principal Findings:**

We identified nutrient patterns from food frequency questionnaires (FFQ) in the European Prospective Investigation into Cancer and Nutrition (EPIC) Study and used 24-hour dietary recall (24-HDR) data to validate and describe the nutrient patterns and their related food sources. Associations between lifestyle factors and the nutrient patterns were also examined. Principal component analysis (PCA) was applied on 23 nutrients derived from country-specific FFQ combining data from all EPIC centers (N = 477,312). Harmonized 24-HDRs available for a representative sample of the EPIC populations (N = 34,436) provided accurate mean group estimates of nutrients and foods by quintiles of pattern scores, presented graphically. An overall PCA combining all data captured a good proportion of the variance explained in each EPIC center. Four nutrient patterns were identified explaining 67% of the total variance: Principle component (PC) 1 was characterized by a high contribution of nutrients from plant food sources and a low contribution of nutrients from animal food sources; PC2 by a high contribution of micro-nutrients and proteins; PC3 was characterized by polyunsaturated fatty acids and vitamin D; PC4 was characterized by calcium, proteins, riboflavin, and phosphorus. The nutrients with high loadings on a particular pattern as derived from country-specific FFQ also showed high deviations in their mean EPIC intakes by quintiles of pattern scores when estimated from 24-HDR. Center and energy intake explained most of the variability in pattern scores.

**Conclusion/Significance:**

The use of 24-HDR enabled internal validation and facilitated the interpretation of the nutrient patterns derived from FFQs in term of food sources. These outcomes open research opportunities and perspectives of using nutrient patterns in future studies particularly at international level.

## Introduction

Dietary pattern analyses are a complementary strategy to the traditional single-food or nutrient approach for capturing the intrinsic complexity of diet, the inter-relationships between its different components and the heterogeneity in food and nutrient patterns existing within and between populations [1], [2]. Exploratory dimension reduction methods have been increasingly used to derive empirical dietary patterns (using principal components analysis or factor analysis) and enabled the identification of dietary patterns, e.g. “Western”, “Mediterranean” or “Prudent” diet, which are potentially associated with different chronic diseases, including cancer [2]–[5]. These multivariate approaches aim to summarize a large number of correlated dietary variables (foods, food groups, nutrients or biomarkers) into fewer independent components explaining most of the dietary variability despite large within- and between-subject variations [2], [6]–[8].

Compared with food patterns analyses, limited work has been done on nutrient pattern analyses to date [9]–[22]. Although results from pattern analyses conducted on foods are easier to translate into public health recommendations [23], [24], nutrient patterns studies have several advantages particularly in an international study context. Firstly, nutrients are to a large extent universal, functionally not exchangeable and, in contrast to food patterns, may characterize specific nutritional profiles in a more easy way to compare populations. Additionally, unlike foods, nutrients show a limited number of non-consumers [25]. These specific features facilitate the statistical analyses, interpretation and generalization of nutrient patterns across populations. Furthermore, the nutrient pattern approach could better mirror a combination of bioactive nutrients in complex biological mechanisms associated with diseases as compared to the use of food patterns [11]–[21], [26]. Finally, recent research emphasizes the use of nutritional biomarkers and metabolites in epidemiological studies [8], [27], [28] and nutrient patterns act as an interface between food patterns and the food metabolome integrating measurements of both diet and metabolism [29].

Among the studies on nutrient patterns available [11]–[18], [20], [26], [30], only one study has been performed at an international level [21]. This may be because of a lack in both standardized dietary methods and nutrient databases, and due to specific methodological issues in collecting, analyzing and interpreting dietary data and its association with disease [21], [31].

The aim of this study was to identify nutrient patterns in one of the largest cohort studies on diet and cancer and other non-communicable diseases, the European Prospective Investigation into Cancer and Nutrition cohort (EPIC), combining food frequency questionnaire (FFQ) data from 10 countries. In addition, we used 24-hour dietary recall (24-HDR) data for internal validation of the identified nutrient patterns using Food Frequency Questionnaires (FFQ), to interpret them and illustrate their related food-sources across countries. Associations between socio-demographic and lifestyle factors with these nutrient patterns were also examined.

## Methods

### Study Population

The EPIC study is a multi-center prospective cohort study designed to investigate the associations between diet, cancer and other chronic diseases across 10 European countries: Denmark, France, Germany, Greece, Italy, the Netherlands, Norway, Spain, Sweden, and the United Kingdom [32], [33]. Participants were recruited between 1992 and 1998, and include 521,330 healthy men and women aged 35–70 years from 23 administrative EPIC centers according to different geographical areas, regions and towns. Exceptions were for France (health insurance members), Utrecht (The Netherlands) and Florence (Italy) (participants of Breast Cancer screening programmes), Oxford (United Kingdom) (mostly vegetarian volunteers), and some centers in Spain and Italy (mostly blood donors). The French, Naples (Italy) and Norwegian cohorts were composed only of female participants. Comprehensive details of the methods of recruitment and study design have been published elsewhere [31], [33], [34].

### Measurement of Diet, Lifestyle Factors, Education and Height and Weight

Usual diet was assessed for each individual at recruitment using country-specific and validated dietary questionnaires [31]. Different types of validated country-specific questionnaires were used to capture country-specific food habits: (1) *self-administered quantitative dietary questionnaires* in Northern Italy, The Netherlands, Germany and Greece; (2) s*emi-quantitative food-frequency questionnaires* (FFQs) (with the same standard portion(s) assigned to all subjects) were used in Denmark, Norway, Naples in Italy and Umea in Sweden, United Kingdom; and (3) *Combined dietary methods* were used in Malmo (Sweden), combining a short non-quantitative food-frequency questionnaire with a 14-day record on hot meals (lunches and dinners). We refer to these questionnaires as baseline country-specific FFQs.

In addition, a single 24-HDR was collected between 1995 and 2000 using EPIC-Soft (IARC, Lyon, France) specially designed to standardize the recall interviews [35]. The 24-HDRs are used as reference measurements and were collected from a stratified sample of 36,900 EPIC participants -the Calibration Study- a random sample of 5–12% (United Kingdom 1.5%) obtained from each of the EPIC cohorts [35], [36]. The 24-HDR are used as reference measurements and provide accurate mean estimates of nutrient and foods at the population level [37]. More details on the rationale and characteristics of the calibration study are given elsewhere [34], [36]–[38]. The 24-HDRs were collected by trained personnel in a face-to-face interview, except in Norway where it was collected by telephone. Food portion sizes were estimated with a common picture book and other methods including standard units and household measures. The interviews were distributed over season and days of the week [36]. All foods were classified according to the common EPIC-Soft food classification as described elsewhere [38].

Individual intakes of 23 nutrients, water, alcohol and total energy were estimated from the baseline country-specific FFQs and the 24-HDRs data using a common food composition database standardised across the countries involved in EPIC (EPIC Nutrient Database, ENDB), recently enriched with folate data [39], [40]. Supplement use were not included in the calculation of nutrient intakes.

Information on physical activity, history of tobacco smoking, alcohol consumption, and education was collected at baseline by questionnaires. Weight and height were self-reported in most centers by the participants during the 24-HDR interview [36].

### Exclusion Criteria

Among the 521,330 EPIC participants, 6,902 subjects were excluded from the pattern analysis because they had missing baseline dietary questionnaires. To prevent inclusion of extreme values, 10,241 subjects were excluded because they were in the lowest and highest first percent of the distribution of the ratio of reported total energy intake to energy requirement. Additionally 22,432 participants were excluded because they had a prevalent cancer at any site at baseline other than non-melanoma skin cancer or were lost during the follow-up as well as 4,443 participants with missing information on lifestyle factors. These exclusions are made to be consistent with those applied in EPIC diet-disease association studies. Statistical pattern analyses were conducted on 477,312 participants, including 34,436 participants from the Calibration Study with 24-HDRs.

### Statistical Analysis

Nutrient pattern analyses were performed using Principal Component Analysis (PCA) [41] based on the combined, but country-specific FFQ derived intake of 23 nutrients. We refer to this as an ‘overall PCA’. Total fat was divided into monounsaturated, polyunsaturated, saturated fatty acids and cholesterol, whilst total available carbohydrates were divided into starch and sugars (monosaccharides and disaccharides). Alcohol consumption was considered as a main lifestyle factor and was not included in the initial list of variables to derive nutrient patterns as reported elsewhere [15], [42], [43]. Besides, when alcohol was included in the analysis, alcohol was the only variable that contributed to the first pattern defined and was found to be only weakly dependent on other nutrients (Pearson correlation coefficients (log scale) of alcohol ranged from |r| = –0.13 with sugar to |r| = 0.03 with magnesium; all correlations were statistically significant).

Variables were log transformed (natural log) after comparing various analysis options with regard to proportion of variance captured. Log transformation also renders the variances and covariances independent of scale. PCA was used with the covariance matrix, rather than the correlation matrix. While the correlation matrix is often used in the epidemiology literature, this is not strictly PCA [44] and the justification of bringing all measures on the same scale is irrelevant after log-transformation.

In order to capture variability of nutrient intakes independently from variation in energy intake, nutrients (log variables) were adjusted for alcohol-free energy before applying PCA using the nutrient density method [45]. We did not adjust for ‘Center/country’ because our objective was to ascertain patterns across Europe rather than within study centers. PCA were conducted on both sexes combined and separated. As comparable patterns were observed in both sexes in PCA without alcohol included, the final results are presented for both sexes combined. The number of the retained principal components (PC) or “patterns” was determined taking into account the interpretation of the patterns, the percentage of total variance explained and the visual inflections in the scree-plots of eigen-values [41]. The loadings represent covariance between the nutrients and the patterns. Nutrients with positive loadings were positively associated with a nutrient pattern while negative loadings are inversely associated. Individual PC scores were then computed from each retained pattern as the sum of products of the observed variables (nutrient intakes [g/day] multiplied by weights proportional to the nutrient’s loading on the pattern [41]. The scores had means of 0 but are not standardized to unit variance to keep their original variances (corresponding to their eigenvalues).

#### Comparison between centers

Separate PCA were carried out on the same variables by country and center and the results were compared to the overall PCA. We aimed to calculate the proportion of variance captured by k center-specific PCs which is also captured by the PCs from the overall PCA (B_k_), in other words how much the center-specific and the overall PCA agreed. Krzanowski’s method was used [46], which is based on the comparison of eigenspaces :
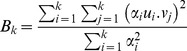



Let u_1_,…,u_k_ and v_1_,…,v_k_ be the PCs resulting from two distinct PCA and u_i_.u_j_ = v_i_.v_j_ = 0 for all i≠j, and u_i_.u_i_ = v_i_.v_i_ = 1; 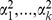 are the eigenvalues (variances) corresponding to the PCs u. The B_k_ measure the proportion of variance in the u-frame which is retained when changing to the v-frame.

**Figure 1:**
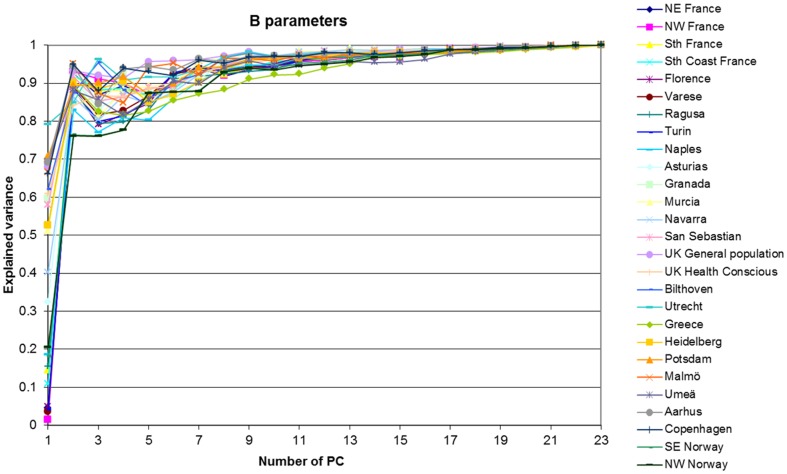
Proportion of the variance in each EPIC center captured in an overall PCA on combined data by the number of PC retained.

The overall PCA, combining data from all EPIC centers, allows capturing a good proportion of the variance explained in each EPIC center (Figure 1). Note that since the first two eigenvalues are relatively close, it can occur that the order of the first two PC’s can change between centers. Hence agreement in the first PC was low in some centers (B_1_<0∶10 for 4 centers), but good when at least the first two components were combined. More than 75% of the variance that would be captured by center-specific PCs was captured by the PCs from the overall PCA (B_j_>0∶76 for all j≥2, B_2_>0∶85 for 23 of 27 centers). Retaining 4 or more PCs was sufficient to capture at least 80% of variance in any center (B_j_>0∶80 for all j≥4). We conducted similar analyses to study sex differences and the difference between genders in each center was quite small provided k>2 (Figure not shown). With 23 centers from 10 countries, EPIC accounts for a wide heterogeneity in diet [25], [47].

#### Description of nutrient patterns combining FFQ and 24-HDR measurements

This analysis was performed on the 34,436 participants in the Calibration Study. We classified the participants into 5 categories based on the quintiles of each PC score. The 24-HDR mean intake for the *i*th nutrient, food or food group, m(*i*), was calculated for participants in each quintile of the PC scores. A generalized linear regression model was used to estimate means adjusted for age, sex, height, weight, country/center and total energy intake to correct for physiological differences of the participants across the EPIC centers/countries. Models were weighted for seasons and days of the week of recall to control for differences in sampling procedures of the 24-HDR interviews [36]. Overall “EPIC mean” intake, M(i), was also calculated for the same nutrient, food or food groups, as the mean in the Calibration Study. To express differences between mean intakes of the participants in each quintile category of PC scores and the overall EPIC mean, the deviation of the nutrient or food intake relative to the EPIC mean was calculated for each nutrient/food, as: 100%* [m(i)/M(i)].

A multi-dimensional “radar” graphic presentation of the relative nutrient and food intakes was used to illustrate contrasts in nutrient, food or food group intakes by quintiles of PC scores. EPIC means, used as the common denominator to calculate deviations, are indicated in each figure by a reference circle at 100% and a range of 0–150%. If the relative consumption of a nutrient/food is above 100%, it indicates that the given quintile of PC score is characterized by a relatively high consumption of that nutrient/food compared with the reference EPIC mean, and vice versa when the relative intake is below 100%. The end peaks of means exceeding 150% are not reported in the graphs but are indicated in Tables S4, S5, S6, S7, S8, S9, S10, S11.

#### Association of nutrient pattern scores with demographic and lifestyle factors

Multiple linear regression models were fitted for each of the PC scores on socio-demographic and lifestyle characteristics at baseline: sex, age at recruitment (per 10 years, continuous), BMI (continuous), log of total energy intake (continuous), physical activity (by category: inactive, moderately inactive, moderately active, active, unknown), smoking status (by category: never, past, current smoker, unknown) educational level (by category: none, primary school completed, technical/professional school completed, secondary school completed, longer education including university degree, not specified) and country/center. The EPIC centers within a country were aggregated at country level to reflect geographical regions that are presumed to share common diets. In contrast, the UK participants were divided into two “general population” (Cambridge and Oxford center) and “health-conscious” (Oxford center, cohort of vegans and ovo-lacto vegetarians) participants [48]. In all models, Spain was chosen as the reference country as its dietary habits depict features of both northern and southern European patterns. We present the regression coefficients and their standard errors. Statistical significance was defined using a 2-sided P-value<0.05. Partial R^2^ were calculated to express the proportion of variance of PC scores explained by each of the measured lifestyle variables given the other independent variables in the model. For this analysis, PC scores were standardized to have a variance of 1. All analyses were performed using SAS software 9.3.

## Results

### Identification of the Nutrient Patterns (PC)

Four nutrient patterns (or PC) were retained by the overall PCA (N = 477,312 participants) and explained about 67% of the total variance (total nutrient variability) (Table 1). Eigenvectors and eigenvalues are presented in Table S1, available online. The 1^st^ PC identified had the largest negative loadings on saturated fatty acids, cholesterol, vitamin B_12_, retinol, and vitamin D (all nutrients of animal origin) and positive loadings for dietary fibre, vitamin C, beta-carotene and folate (nutrients from plant sources, except for folate which has a dominant plant but also animal origin). This pattern accounted for 29% of variance in nutrient intakes.

**Table 1:** Loading matrix and explained variances for the first four Principal Components (PC) identified by PCA*.

Nutrient variables	PC1	PC2	PC3	PC4
Total proteins	−0.10	0.41	0.08	0.55
Saturated Fatty Acids (SFA)	−0.48	0.05	−0.32	−0.18
Monounsaturated Fatty Acids (MUFA)	−0.06	−0.12	−0.24	−0.12
Polyunsaturated Fatty Acids (PUFA)	0.09	0.25	0.26	−0.37
Cholesterol	−0.57	0.30	−0.17	0.25
Starch	−0.05	−0.35	0.22	−0.15
Sugars	0.30	0.14	0.02	0.15
Dietary fibre	0.57	0.33	0.26	−0.04
Thiamin	0.32	0.43	0.32	0.22
Riboflavin	0.06	0.60	−0.12	0.51
Vitamin B_6_	0.37	0.51	0.25	0.36
Folate (Vitamin B_9_)	0.59	0.59	0.03	0.16
Vitamin B_12_	−0.57	0.54	−0.20	0.39
Vitamin C	0.66	0.42	−0.02	0.11
Beta-carotene (β-carotene)	0.60	0.66	−0.12	−0.27
Retinol	−0.73	0.48	−0.26	−0.26
Vitamin E	0.41	0.28	0.10	−0.35
Vitamin D	−0.55	0.41	0.70	−0.06
Calcium	0.14	0.35	−0.16	0.45
Phosphorus	0.11	0.49	0.06	0.48
Iron	0.34	0.34	0.00	0.17
Potassium	0.42	0.59	0.21	0.36
Magnesium	0.30	0.47	0.15	0.23
Proportion of explained variance (%)	29.2	21.8	9.0	7.3
Cumulative explained variance (%)	29.2	51.0	60.0	67.3

*Estimates from a EPIC-Wide PCA done on the country-specific FFQ derived intake levels of 23 nutrients (log-transformed and energy adjusted using the energy density method, using Alcohol-free Energy).

The 2^nd^ PC had the greatest positive loadings on vitamin B complex (specifically riboflavin, B_6_, folate, B_12_), vitamin C, beta-carotene, retinol, phosphorus, potassium and magnesium and negative loading on starch. This pattern accounted for 22% of the variance.

The 3^rd^ PC accounted for 9% of the variance. Vitamin D had the greatest loading of 0.7. Other nutrients contributing to a lesser extent included PUFA, thiamin, Vitamin B_6_ and fibre with positive loadings and SFA and retinol with negative loadings.

The 4^th^ and last PC retained accounted for 7% of the variance and had the greatest positive loadings on calcium, total proteins, riboflavin, and phosphorus and negative loadings on PUFA and Vitamin E.

### Description of the Identified Nutrient Patterns Based on 24-HDR Data

**Figure 2:**
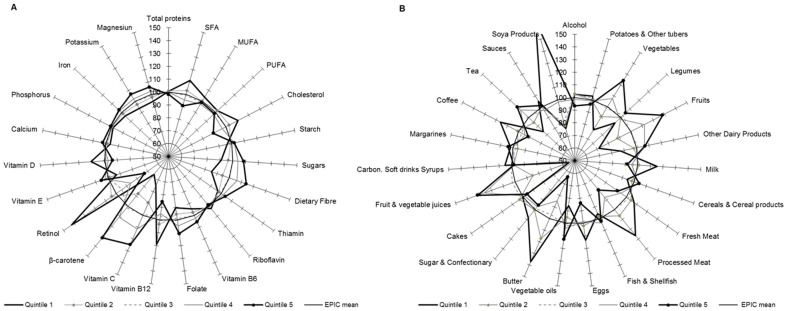
Deviation (%) of the 24-HDR mean intakes from the overall EPIC means among participants in the quintiles of PC1 scores for nutrients (A) and foods (B). Means are adjusted for age, sex, height, weight and energy and weighted for day and season of recall (N = 34,436). The reference circle of the radius (100%) correspond to the ‘EPIC means’ and the spikes indicate the deviation of the specific nutrient mean in quintiles of pattern scores from the reference ‘EPIC means’.

**Figure 3:**
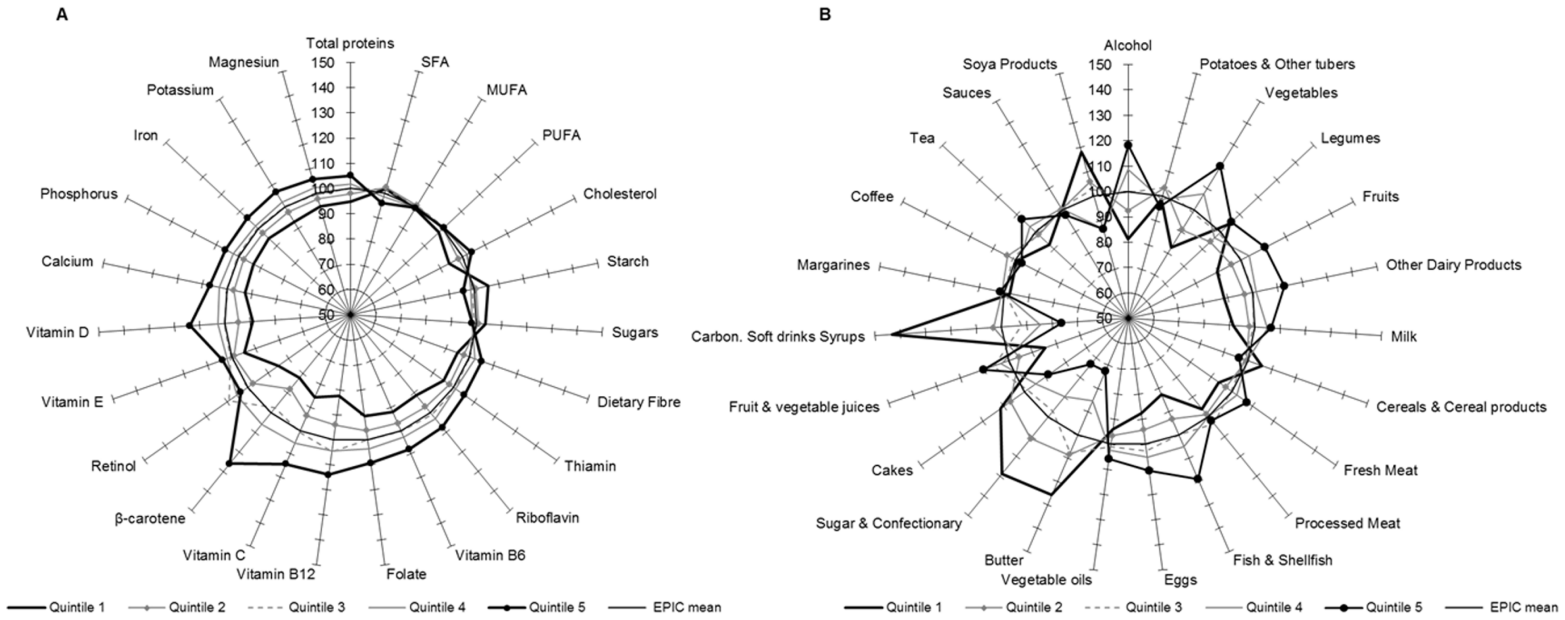
Deviation (%) of the 24-HDR mean intakes from the overall ‘EPIC means’ among participants in the quintiles of PC2 scores for nutrients (A) and foods (B). Means are adjusted for age, sex, height, weight and energy and weighted for day and season of recall (N = 34,436). The reference circle of the radius (100%) correspond to the ‘EPIC means’ and the spikes indicate the deviation of the specific nutrient mean in quintiles of pattern scores from the reference ‘EPIC means’.

**Figure 4:**
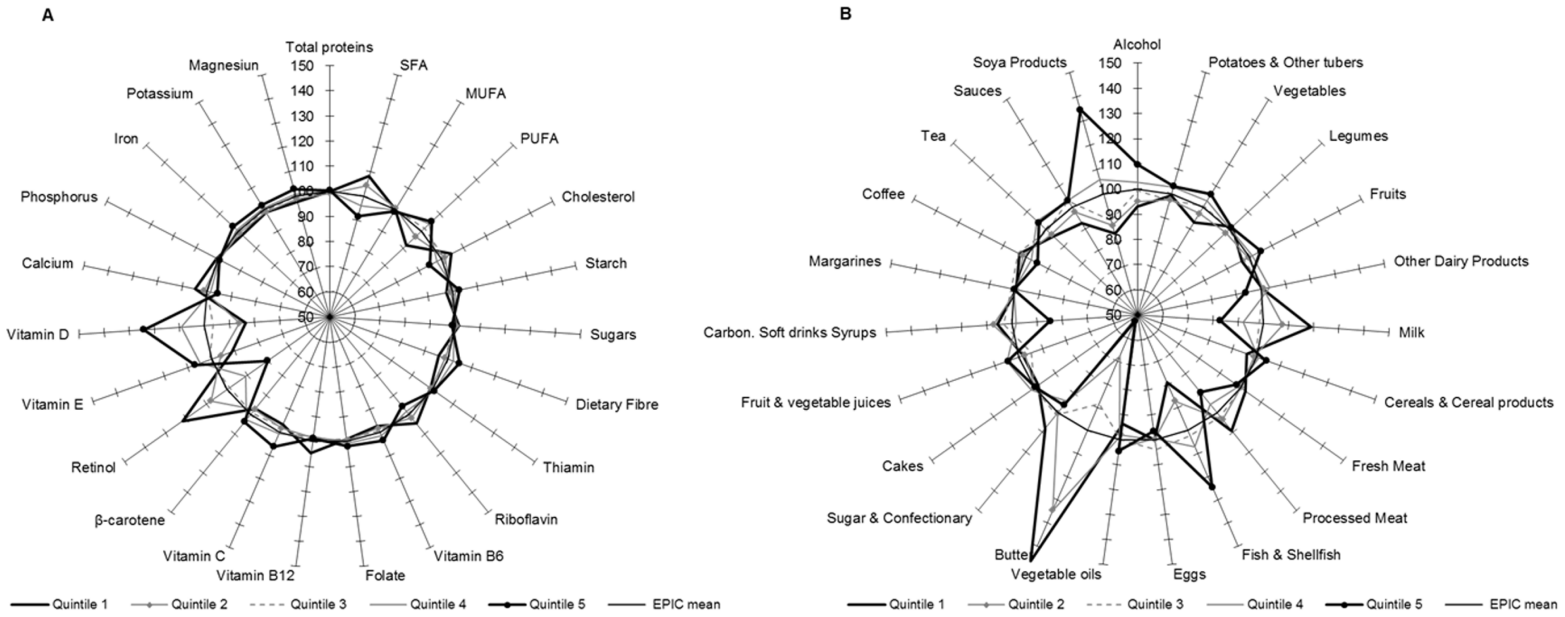
Deviation (%) of the 24-HDR mean intakes from the overall ‘EPIC means’ among participants in the quintiles of PC3 scores for nutrients (A) and foods (B). Means are adjusted for age, sex, height, weight and energy and weighted for day and season of recall (N = 34,436). The reference circle of the radius (100%) correspond to the ‘EPIC means’ and the spikes indicate the deviation of the specific nutrient mean in quintiles of pattern scores from the reference ‘EPIC means’.

Figure 2, 3, 4, 5 show graphically the deviations of the adjusted 24-HDR mean intake of nutrients and foods/food groups by different quintiles of PC scores relative to their respective nutrient and foods/food group overall EPIC mean intake. Corresponding numbers and their deviations are presented online in Tables S4, S5, S6, S7, S8, S9, S10, S11. The nutrients with high loadings on a particular pattern (table 1) also showed high deviations in their mean intakes from the overall EPIC means by quintiles of pattern scores as estimated from standardized 24-HDR.

**Figure 5:**
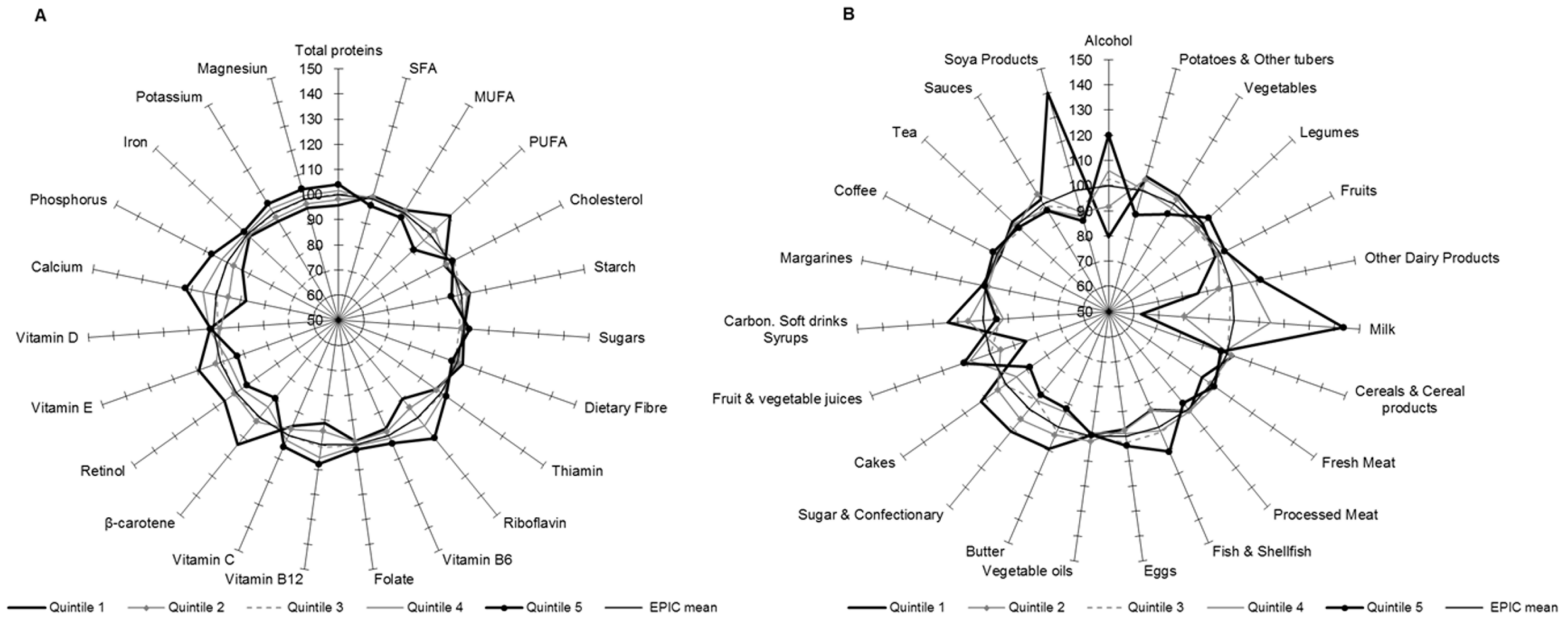
Deviation (%) of the 24-HDR mean intakes from the overall ‘EPIC means’ among participants in the quintiles of PC4 scores for nutrients (A) and foods (B). Means are adjusted for age, sex, height, weight and energy and weighted for day and season of recall (N = 34,436). The reference circle of the radius (100%) correspond to the ‘EPIC means’ and the spikes indicate the deviation of the specific nutrient mean in quintiles of pattern scores from the reference ‘EPIC means’.

#### PC1

In comparison with the overall EPIC mean, participants in the 1^st^ quintile of PC1 score were characterized by high intakes of SFA, cholesterol, vitamin B_12_, vitamin D and retinol in contrast to low intakes of dietary fibre, vitamin C and beta carotene. When compared to the EPIC means, participants in the 5^th^ quintile of score reported opposite associations (Figure 2; table S4). When considering their related food contributions, animal based foods dominated in the 1^st^ quintile including meat, processed meat, butter, eggs and also coffee (Figure 2; Table S8). Mean intakes of plant foods in the 1^st^ quintile were lower than the EPIC means. In contrast, participants in the 5^th^ quintile were characterized by a diet richer in plant foods (fruits, vegetables, fruit juices, soya products, vegetable oils and tea) and lower in animal food intakes, in comparison with the overall EPIC mean.

#### PC2

In the 1^st^ quintile of PC2 score, intakes of vitamins B_6_, B_12_, Folate, riboflavin, vitamin C, beta-carotene, retinol, phosphorus, potassium and magnesium were relatively low in comparison with the overall EPIC mean, whereas they were high in the 5^th^ quintile (Figure 3; Table S5). Participants in the 5^th^ quintile of score have a diet rich in fruits, vegetables, fresh meat, eggs, fish and tea, but low consumption of soft drinks, cakes, sugar and butter relative to the EPIC means (Figure 3; Table S9).

#### PC3

A high mean intake of Vitamin D and PUFA was observed in the 5^th^ quintile of score, higher than the EPIC mean by respectively 24% and 5%, while in the 1^st^ quintile the mean intake was respectively 16% and 8% below the EPIC mean (Figure 4; Table S6). Regarding the food consumption, participants in the 5^th^ quintile of score had a diet with a higher consumption of fish and soya products but also oils, fruits and vegetables and cereals in comparison with the EPIC means. Fish and soy product intakes in this quintile were respectively 24% and 35% higher than the EPIC mean, while respectively 21% and 16% lower in the 1^st^ quintile (Figure 4; Table S10).

#### PC4

In comparison with the EPIC means, this pattern was characterized by high intakes of PUFA, beta carotene, retinol and vitamin E in the 1^st^ quintile, with corresponding low intakes in the 5^th^ quintile. Calcium, Vitamin B_12_, Riboflavin, phosphorus, potassium and total protein intakes were much lower in the 1^st^ quintile and higher than the EPIC mean by up to 12% in the 5^th^ quintile. In terms of foods, dairy product consumption, especially milk, increases from the 1^st^ to 5^th^ quintile, while soy products had high consumption in the 1^st^ quintile. Besides, intake of fish was relatively high in the 5^th^ quintile (Figure 5; Table S7 and S11).

### Demographic and Lifestyle Factors Associated with the Identified Nutrient Patterns

Tables 2 and 3 show the regression coefficients and partial R-squared of individual PC scores for each of the four patterns retained for demographic and lifestyle factors, country of recruitment and energy intake. Corresponding mean values of baseline factors by PC quintile are presented in Table S2. Country and total energy intake were the most important measured predictors for the four retained PC scores (Table 3). Country accounted for more than 12% of the variability of each PC, with the least contribution to PC4 (12%) and the greatest to PC1 (24%). Distribution of participants by country and quintiles of pattern scores are presented in Table S3. Variability attributable to total energy ranged from 1% (PC3) to 4% (PC1).

**Table 2:** Multiple regression derived coefficients (β) and standard errors (SE) of specified predictors for the four nutrient pattern scores*.

Variables	PC1			PC2			PC3			PC4		
	β	SE	P value	β	SE	P value	β	SE	P value	β	SE	P value
Age†	0.015	0.001	<0.001	0.096	0.001	<0.001	0.012	0.001	<0.001	0.03	0.002	<0.001
BMI	−0.008	0.0003	<0.001	0.016	0.0003	<0.001	0.001	0.0003	<0.001	0.017	0.0003	<0.001
Log (Energy)‡	−6.258	0.042	<0.001	−6.195	0.047	<0.001	−3.078	0.0435	<0.001	−6.606	0.049	<0.001
Sex												
Female	0.2	0.003	<0.001	0.307	0.003	<0.001	−0.122	0.003	<0.001	−0.06	0.003	<0.001
Male	reference			reference			reference			reference		
Smoking status												
Former	0.016	0.003	<0.001	0.072	0.003	<0.001	0.028	0.003	<0.001	0.044	0.003	<0.001
Smoker	−0.189	0.003	<0.001	0.006	0.003	0.061	−0.045	0.003	<0.001	0.088	0.003	<0.001
Unknown	−0.013	0.008	0.112	0.067	0.009	<0.001	0.022	0.008	0.009	0.058	0.01	<0.001
Never	reference		reference				reference			reference		
Educational level												
Longer education	0.079	0.007	<0.001	0.457	0.008	<0.001	0.018	0.007	0.014	0.089	0.008	<0.001
Not specified	−0.07	0.009	<0.001	0.423	0.01	<0.001	0.004	0.009	0.685	0.093	0.011	<0.001
Primary school completed	−0.063	0.007	<0.001	0.257	0.007	<0.001	−0.004	0.007	0.589	0.061	0.008	<0.001
Secondary school	0.037	0.007	<0.001	0.414	0.008	<0.001	0.012	0.007	0.091	0.07	0.008	<0.001
Technical/professional school	0.003	0.007	0.635	0.381	0.008	<0.001	0.029	0.007	<0.001	0.07	0.008	<0.001
None	reference			reference			reference			reference		
Physical activity												
Active	0.061	0.005	<0.001	0.103	0.005	<0.001	−0.009	0.005	0.063	0.009	0.005	0.084
Inactive	−0.037	0.004	<0.001	0.001	0.004	0.879	0.006	0.004	0.097	0.021	0.004	<0.001
Missing	0.071	0.006	<0.001	−0.153	0.007	<0.001	0.012	0.006	0.056	−0.196	0.007	<0.001
Moderately active	0.018	0.003	<0.001	0.057	0.003	<0.001	−0.008	0.003	0.01	−0.009	0.003	0.013
Moderately inactive	reference			reference			reference			reference		
Participating countries/centers												
Denmark	−0.782	0.006	<0.001	0.74	0.006	<0.001	−0.676	0.006	<0.001	−0.569	0.007	<0.001
France	−0.236	0.006	<0.001	0.682	0.007	<0.001	−1.219	0.006	<0.001	−0.857	0.007	<0.001
Germany	−0.593	0.006	<0.001	0.3	0.006	<0.001	−0.628	0.006	<0.001	−1.278	0.007	<0.001
Greece	0.974	0.006	<0.001	0.225	0.007	<0.001	−1.302	0.006	<0.001	−1.395	0.007	<0.001
Italy	−0.34	0.006	<0.001	−0.388	0.006	<0.001	−1.195	0.006	<0.001	−0.707	0.007	<0.001
Norway	−1.245	0.009	<0.001	0.814	0.01	<0.001	0.603	0.009	<0.001	−1.338	0.01	<0.001
Sweden	−1.228	0.007	<0.001	0.275	0.007	<0.001	0.094	0.007	<0.001	−1.074	0.008	<0.001
The Netherlands	−0.287	0.006	<0.001	0.002	0.007	0.79	−0.573	0.006	<0.001	−1.119	0.007	<0.001
UK General population	−0.18	0.007	<0.001	0.831	0.007	<0.001	−0.239	0.007	<0.001	−0.522	0.008	<0.001
UK Health conscious	0.456	0.006	<0.001	0.637	0.007	<0.001	−0.052	0.006	<0.001	−0.861	0.007	<0.001
Spain	reference			reference			reference			reference		

*he scores had means of 0 but are standardized to unit variance; PC scores calculated on the country-specific FFQ derived intake levels of 23 nutrients, n = 477,312.

† 10 years increase.

‡ 10 units increase.

**Table 3:** P-values of F-test on type III sum of squares estimate.

Variable		PC1			PC2			PC3			PC4		
	DF#	Partial R^2^	%	P value	Partial R^2^	%	P value	Partial R^2^	%	P value	Partial R^2^	%	P value
Log(Energy)†	1	0.043	4.35	<0.001	0.0353	3.53	<0.001	0.010	1.04	<0.001	0.037	3.66	<0.001
Age‡	1	0.0002	0.02	<0.001	0.0086	0.86	<0.001	0.0001	0.01	<0.001	0.001	0.08	<0.001
BMI	1	0.0016	0.16	<0.001	0.0052	0.52	<0.001	0.00005	0.01	<0.001	0.005	0.55	<0.001
Smoking Status	3	0.0100	1.00	<0.001	0.0013	0.13	<0.001	0.001	0.10	<0.001	0.001	0.14	<0.001
Sex	1	0.010	0.96	<0.001	0.0184	1.84	<0.001	0.003	0.34	<0.001	0.001	0.07	<0.001
Education level	5	0.0038	0.38	<0.001	0.0098	0.98	<0.001	0.0002	0.02	<0.001	0.0003	0.03	<0.001
Physical activity	4	0.0012	0.12	<0.001	0.003	0.28	<0.001	0.00005	0.005	<0.001	0.002	0.18	<0.001
Country/Center	10	0.2478	24.78	<0.001	0.1351	13.51	<0.001	0.237	23.68	<0.001	0.122	12.20	<0.001

*The scores had means of 0 but are standardized to unit variance; PC scores calculated on the country-specific FFQ derived intake levels of 23 nutrients, n = 477,312.

† 10 units increase.

‡ 10 years increase.

# Degree of Freedom.

Study participants with high scores on PC1 were more likely to be female, had a higher education, were more often former smokers and less frequently current smokers, had a higher level of physical activity, were older, had lower energy intake, and a lower BMI than participants with lower scores. Participants living in Greece and the UK health conscious had higher overall scores as compared to Spain (referent category). The remaining countries had lower scores (Table 2).

Participants with high scores on PC2 were more likely to be female, former smokers, more well educated and physically active and with lower total energy intake. As compared to Spain, participants from the rest of the countries in the cohort had higher scores with the exception of participants from Italy. The socio-demographic characteristics of individuals in PC1 and PC2 with higher scores were relatively similar. PC3 score was positively associated with age, BMI and former smoking and was inversely associated with female sex, lower education, lower levels of physical activity, current smoking and total energy intake. The Nordic countries (Norway and Sweden) had the highest scores followed by Spain. PC4 *score* was positively associated with age, BMI, higher education, smoking (both smokers and former smokers) but negatively associated with female sex and total energy intake. As compared to Spain, all the other countries had lower scores (Table 2).

## Discussion

We identified four nutrient patterns using PCA across the 23 European centers participating in the EPIC study. We showed the applicability of an overall PCA combining all data since nutrient patterns revealed themselves to be reproducible across EPIC centers. We then used the standardized 24-HDRs collected in a representative sub-sample of the EPIC study to describe these patterns and depict their related food sources. The use of 24-HDR allowed internal validation of the patterns obtained using the FFQ data: the 24-HDRs provide good mean estimates at the population level in a comparable way across countries [48]. Our analysis was therefore focused on the comparison of mean dietary intakes within each quintile of pattern scores. Additionally, we investigated the relationship between the nutrient patterns and socio-demographic and lifestyle characteristics of the participants.

For this nutrient pattern analysis, we benefit from the unique features of the EPIC cohort, involving a European study population with a large geographical spread and high heterogeneity in dietary intakes and patterns [31]. The EPIC study offered the ideal setting to address a series of methodological challenges such as normalisation, transformation and scaling of variables, energy adjustment, how to deal with heterogeneous data between centers and sexes to implement dimension reduction methods such as PCA. The EPIC study also offered the opportunity to use two complementary dietary assessment methods (FFQ and 24-HDRs) to identify and describe the patterns. The internal approach has been used in the Framingham Study to describe clusters defined on FFQs data with mean intakes of nutrients derived from an independent 3-day food record [49].

All studies published so far on nutrient patterns were conducted at the national level in different geographic areas and populations, except one combining data from 5 case-control studies [21]. These previous studies consistently identified a nutrient pattern labeled as “meat” [10], [19], “high-meat” [13], [18], “animal products” [9], [11], [15], [16] or “animal products and cereals” [21], which was characterized by nutrients from animal food sources. In our study we identified a pattern characterized by positive loadings of nutrients essentially from plant food sources and negative loadings of nutrients that tend to be correlated at the individual level with animal food sources. Second, previous studies have also consistently identified a nutrient pattern labelled as “fiber and vitamins” [9], [11], [15]–[17], [20], [22], [30] or “vitamins-rich” [14] or “antioxidant vitamins and fiber” [21], characterized by a diet rich in vitamins and minerals and sharing similar features with our 2^nd^ pattern with high loadings on a number of micro-nutrients and proteins. Our PC3 has similar features with the “polyunsaturated fatty acids and vitamin D” pattern reported elsewhere [22], with high loadings on Vitamin D and PUFAs.

Compared to foods, nutrients are to a large extent universal and are absorbed, although with some variability, whatever the food consumed, and functionally not exchangeable. In contrast to food patterns, nutrient patterns may characterize specific nutritional profiles in a more easy way to compare populations. This approach is particularly useful to identify combinations of nutrients that could reflect possible biological mechanisms. Despite the heterogeneity in the foods consumed within and between individuals and study populations in the EPIC cohort [48], PC1 and PC2 were driven by nutrients that can be found in many food groups and were therefore independent from the food groups they came from. They reflect a broad range of food sources and thus the most prevalent types of dietary patterns which explain the largest proportion of the variance (51%). In contrast, the 3^rd^ and the 4^th^ patterns are more related to specific food sources were variation is less pronounced i.e. fish and soy products for PC3 (high contribution of vitamin D and PUFA) and milk for PC4 (high contribution of calcium, phosphorus, proteins, riboflavin).

The first four PCs retained in our analysis explained a high proportion of the total variance in the original data (67%), higher than those reported in food pattern analysis: the percentage of variance explained by the first PC is relatively high when compared to that reported in studies of dietary patterns on the same data defined using similar methods [50], [51]. This is probably due to the use of nutrients rather than using foods or food groups as variables in the multivariate analyses [15]. The percentage of explained variance in our study is comparable to that reported in other studies on nutrient-based patterns.

In this study, nutrient intakes were derived from the usual food consumption data collected through country-specific FFQs which are prone to measurement errors and potentially introduce systematic between-country differences in nutrient assessment. The number of questions related to consumption of specific foods was adapted to local customs in the country-specific FFQs because these habits vary between countries [31]. The distribution of quintiles of pattern scores by countries or centers (Table S2) illustrated heterogeneity in diet across EPIC centers already observed and reported before [25], [47]. However, harmonized food composition tables across European countries were used to translate food into nutrient intakes thus sizeably improving the comparability of nutrient intakes [39].

The use of dietary supplements was not included in the calculation of nutrient pattern scores. Previous study has shown some heterogeneity regarding the proportion of dietary supplement users in the EPIC Study, with a high consumption in northern countries [52]. In our analysis, we have depicted nutrient patterns from natural food sources only without having supplements included. Given the limited evidence on the protective and detrimental effects of food supplements, most of the nutritional recommendations and guidelines promote the use of a wide variety of foods above the use of food supplements [53]. In a sensitivity analysis, we have checked whether dietary supplement use (categorical variable: Yes, No, Unknown) contributes to the variability of each PC score, but the contribution was negligible (data not shown).

The EPIC centers were identified to be the main factor explaining the variability in PC scores (partial R2 analysis-Table 3). To capture the variability between the nutritional variables independently of a center effect on dietary measures, one solution would have been to use the consumption of nutrients adjusted for the center by subtracting the average center score, but this would have restrained the nutrient patterns to intra-center variation only. Combined data from all the EPIC centers (without adjustment for ‘center’) was preferred as the main objective was this analysis was to ascertain and compare patterns across Europe rather than within study centers.

Energy intake was the second most important factor explaining variability in PC scores, despite the use of energy density normalization [45] prior to applying PCA. Normalization for total energy helps to remove variation due to body size and metabolic rate [45] and should have contributed to reduce measurement errors in reported dietary intakes and increase nutrient pattern comparability across countries [34]. This does not contradict the possibility that those eating a high energy diet tend to eat a different pattern of foods and hence nutrients.

The use of a PCA approach to define nutrient patterns in this project has advantages as compared to Factor analysis (FA). PCs are generated sequentially, meaning that the variance explained by the first factor is removed and the second factor is then generated to maximally explain the remaining variance. The definition of each factor is independent of the number of factors retained, which is not the case for FA. The PC scores are also orthogonal and the patterns are objective (no use of rotations). Besides, using PCA, nutrients could load on multiple patterns which is not the case with FA. Although PCA complicates the interpretation of the patterns, this approach is particulartly usefull in the context of nutrient patterns in order to identify combination of nutrients that could reflect possible biological mechanisms.

Among the limitations related to the PCA approach are subjective decisions on how to interpret nutrient patterns. There are questions such as the choice of variables to include in the analysis, whether to transform and or standardize the data, the number of components to retain and finally the threshold for factor loadings (i.e. in this analysis |0.45|) [41]. In addition, patterns identified do not provide an immediate picture of exactly what is being consumed, as the same scores may be obtained with different combinations of nutrients or different quantities of foods, which may be high or low in nutrient density. This method can be influenced by the way in which nutrients are grouped, as this may obscure the patterns within subpopulations or artificially separate them based on inter correlations of uniquely consumed foods [54].

A disadvantage of a nutrient-based approach is that nutrients are less directly related to dietary recommendations because ultimately, nutrient intakes are largely determined by the choice of food sources. Since many food sources exist for the same nutrient, it is challenging to make food-based dietary recommendations. However, our study addressed these challenges. Indeed, the integration of standardised 24-HDRs for estimating nutrient intakes from a representative sub-sample of our whole study population enabled both to validate the nutrient patterns as well as to identify their main specific food sources. These results confirm the increasing potentials of integrated dietary approaches, increasingly recommended in nutritional epidemiological studies and stress the need to pursue this still under explored research area [55].

Besides, the use of identified nutrient patterns in examining diet-disease relationships has been questioned [56]: PCA aims at maximising the fraction of variance explained by a weighted linear combination of original variables, but the aspects of nutrition which are most variable need not be those that are most strongly associated with disease. Indeed it could be argued that the most variable aspects of human diet could be those that have least bearing on health. Despite these limitations, the promising and consistent results obtained from this analysis contribute to new knowledge and open new research perspectives.

## Conclusions

This analysis identified four nutrient patterns and the use of two independent and complementary dietary assessment tools (FFQ and standardized 24-HDR) enabled their internal validation and interpretation in a complex international study context. It is anticipated that the proposed approach will facilitate the integration of nutrient patterns into multivariate and multi-level analyses of dietary exposure (incl. food, nutrient and biological/omic patterns) and strengthen the understanding of its association with diseases. In addition, this should open new perspectives in a research domain still under-explored and facilitate internationalization of public health recommendations through a better understanding and integration of nutrient patterns.

## Supporting Information

Table S1 Eigenvectors and corresponding eigen-values of the Covariance Matrix for the first four Principal Components (PC) identified by PCA.
(DOCX)

Table S2 Distribution of non-dietary factors and total energy intake by quintiles of nutrient pattern scores in EPIC.
(DOCX)

Table S3 Distribution of participants by country and quintiles of nutrient pattern scores in EPIC.
(DOCX)

Table S4 Daily mean nutrient intakes in the EPIC Calibration study (EPIC Mean) and per quintiles of PC1 scores and percentage deviation of the quintile mean from the overall EPIC mean.
(DOCX)

Table S5 Daily mean nutrient intakes in the EPIC Calibration study (EPIC Mean) and per quintiles of PC2 scores and percentage deviation of the quintile mean from the overall EPIC mean.
(DOCX)

Table S6 Daily mean nutrient intakes in the EPIC Calibration study (EPIC Mean) and per quintiles of PC3 scores and percentage deviation of the quintile mean from the overall EPIC mean.
(DOCX)

Table S7 Daily mean nutrient intakes in the EPIC Calibration study (EPIC Mean) and per quintiles of PC4 scores and percentage deviation of the quintile mean from the overall EPIC mean.
(DOCX)

Table S8 Daily means of food/food group intakes in the EPIC Calibration study (EPIC Mean) and per quintiles of PC1 scores and percentage deviation of the quintile mean from the overall EPIC mean.
(DOCX)

Table S9 Daily means of food/food group intakes in the EPIC Calibration study (EPIC Mean) and per quintiles of PC2 scores and percentage deviation of the quintile mean from the overall EPIC mean.
(DOCX)

Table S10 Daily means of food/food group intakes in the EPIC Calibration study (EPIC Mean) and per quintiles of PC3 scores and percentage deviation of the quintile mean from the overall EPIC mean.
(DOCX)

Table S11 Daily means of food/food group intakes in the EPIC calibration study (EPIC Mean) and per quintiles of PC4 scores and percentage deviation of the quintile mean from the overall EPIC mean.
(DOCX)
