# Supplementary material for: Nutrient Patterns and Their Food Sources in an International Study Setting: Report from the EPIC Study
Source: PLoS One. 2014 Jun 5;9(6):e98647. doi: 10.1371/journal.pone.0098647 (PMC4047062; doi:10.1371/journal.pone.0098647)
Supplement: Table S1 — Eigenvectors and corresponding eigen-values of the Covariance Matrix for the first four Principal Components (PC) identified by PCA. (DOCX) [file pone.0098647.s001.docx]

**Table S1. Eigenvectors and corresponding eigen-values of the Covariance Matrix for the first four Principal Components (PC) identified by PCA*.**

| Nutrient variable | PC1 | PC2 | PC3 | PC4 |
| --- | --- | --- | --- | --- |
| Total proteins | -0.02 | 0.08 | 0.03 | 0.19 |
| Saturated Fatty Acids (SFA) | -0.12 | 0.02 | -0.15 | -0.09 |
| Monounsaturated Fatty Acids (MUFA) | -0.02 | -0.04 | -0.12 | -0.07 |
| Polyunsaturated Fatty Acids (PUFA) | 0.03 | 0.09 | 0.15 | -0.24 |
| Cholesterol | -0.24 | 0.15 | -0.13 | 0.20 |
| Starch | -0.02 | -0.11 | 0.11 | -0.08 |
| Sugars | 0.10 | 0.05 | 0.01 | 0.09 |
| Dietary fibre | 0.16 | 0.10 | 0.13 | -0.02 |
| Thiamin | 0.09 | 0.14 | 0.16 | 0.12 |
| Riboflavin | 0.02 | 0.21 | -0.06 | 0.30 |
| Vitamin B_6_ | 0.09 | 0.14 | 0.11 | 0.17 |
| Folate (Vitamin B_9_) | 0.18 | 0.20 | 0.02 | 0.10 |
| Vitamin B_12_ | -0.30 | 0.33 | -0.19 | 0.41 |
| Vitamin C | 0.34 | 0.25 | -0.02 | 0.11 |
| Beta-carotene | 0.43 | 0.55 | -0.15 | -0.39 |
| Retinol | -0.54 | 0.41 | -0.35 | -0.38 |
| Vitamin E | 0.15 | 0.12 | 0.06 | -0.25 |
| Vitamin D | -0.35 | 0.30 | 0.81 | -0.08 |
| Calcium | 0.04 | 0.13 | -0.09 | 0.28 |
| Phosphorus | 0.02 | 0.11 | 0.02 | 0.18 |
| Iron | 0.07 | 0.08 | 0.00 | 0.07 |
| Potassium | 0.09 | 0.15 | 0.08 | 0.16 |
| Magnesium | 0.07 | 0.12 | 0.06 | 0.10 |
| Eigenvalues | 0.95 | 0.71 | 0.29 | 0.24 |

*PCA conducted on the country-specific FFQ derived intake levels of 23 nutrients, n=477,312
