# Supplementary material for: Nutrient Patterns and Their Food Sources in an International Study Setting: Report from the EPIC Study
Source: PLoS One. 2014 Jun 5;9(6):e98647. doi: 10.1371/journal.pone.0098647 (PMC4047062; doi:10.1371/journal.pone.0098647)
Supplement: Table S2 — Distribution of non-dietary factors and total energy intake by quintiles of nutrient pattern scores in EPIC. (DOCX) [file pone.0098647.s002.docx]

**Table S2. Distribution of non-dietary factors and total energy intake by quintiles of nutrient pattern scores in EPIC*.**

|  | PC1 | | | | | | PC2 | | | | | |
| --- | --- | --- | --- | --- | --- | --- | --- | --- | --- | --- | --- | --- |
| Baseline characteristics | Quintile1 | Quintile 2 | Quintile 3 | Quintile 4 | Quintile 5 | p-trend^†^ | Quintile1 | Quintile 2 | Quintile 3 | Quintile 4 | Quintile 5 | p-trend^†^ |
| n | 95462 | 95463 | 95462 | 95463 | 95462 |  | 95462 | 95463 | 95462 | 95463 | 95462 |  |
| Age ^‡^ | 51.9;8.9 | 51.2;8.9 | 51.1;9.4 | 51.1;10.0 | 50.6;11.9 | <0.001 | 49.3;10.3 | 50.5;10.1 | 51.2;9.9 | 51.8;9.7 | 53.1;9.1 | <0.001 |
| Weight ^‡^ | 73.6;14.3 | 71.2;13.8 | 69.7;13.3 | 68.3;12.9 | 67.8;12.9 | <0.001 | 71.6;13.8 | 70.8;13.8 | 70.1;13.7 | 69.5;13.5 | 68.8;13.1 | <0.001 |
| Height ^‡^ | 169.3;9.2 | 167.0;8.9 | 165.6;8.6 | 164.4;8.3 | 163.4;8.4 | <0.001 | 166.9;9.9 | 166.5;9.3 | 166.1;8.8 | 165.6;8.4 | 164.7;7.9 | <0.001 |
| log (Energy) ^‡^ | 7.65;0.3 | 7.63;0.3 | 7.60;0.3 | 7.57;0.3 | 7.49;0.3 | <0.001 | 7.7;0.3 | 7.6;0.3 | 7.6;0.3 | 7.5;0.3 | 7.5;0.3 | <0.001 |
| Sex |  |  |  |  |  |  |  |  |  |  |  |  |
| Male % | 46.9 | 32.8 | 25.9 | 21.5 | 22.0 |  | 45.3 | 35.2 | 28.8 | 23.3 | 16.4 | <0.001 |
| Female % | 53.1 | 67.2 | 74.1 | 78.5 | 78.0 | <0.001 | 54.7 | 64.8 | 71.2 | 76.7 | 83.6 | <0.001 |
| Highest school level |  |  |  |  |  |  |  |  |  |  |  |  |
| None-Primary school % | 34.7 | 28.8 | 27.7 | 27.4 | 31.2 | <0.001 | 41.2 | 33.0 | 28.5 | 24.7 | 22.5 | <0.001 |
| Technical- Secondary school % | 44.8 | 45.6 | 44.8 | 42.4 | 35.8 |  | 38.3 | 41.1 | 42.8 | 44.7 | 46.5 |  |
| Longer education (incl. University deg.) % | 19.1 | 22.9 | 23.9 | 25.6 | 27.3 |  | 18.9 | 23.6 | 25.6 | 26.1 | 24.8 |  |
| Physical activity |  |  |  |  |  |  |  |  |  |  |  |  |
| Inactive % | 14.4 | 15.8 | 15.4 | 15.1 | 14.4 |  | 15.3 | 15.0 | 14.9 | 15.1 | 14.9 | 0.027 |
| Moderately inactive % | 24.7 | 28.2 | 30.4 | 33.3 | 33.4 | <0.001 | 27.4 | 28.5 | 29.8 | 31.0 | 33.1 |  |
| Moderately active % | 25.3 | 30.0 | 33.4 | 35.6 | 40.1 |  | 35.6 | 34.6 | 33.0 | 31.7 | 29.5 |  |
| Active % | 7.4 | 7.9 | 8.4 | 8.5 | 9.0 |  | 9.2 | 8.8 | 8.5 | 7.7 | 6.9 |  |
| Smoking status |  |  |  |  |  |  |  |  |  |  |  |  |
| Never smoker % | 37.8 | 45.6 | 49.6 | 53.7 | 57.7 | <0.001 | 46.2 | 48.9 | 50.0 | 50.0 | 49.3 | <0.001 |
| Former smoker % | 27.2 | 27.6 | 27.2 | 26.5 | 24.7 |  | 24.6 | 25.8 | 26.7 | 27.4 | 28.7 |  |
| Smoker % | 33.4 | 25.0 | 21.1 | 17.6 | 15.1 |  | 28.0 | 23.4 | 21.3 | 20.3 | 19.2 |  |

*based on the country-specific FFQ derived intake levels of 23 nutrients, n=477,312

^†^P for trend by Wald test

^‡^mean; SD (standard deviation)

Table S2. continued

|  | PC3 | | | | | | | | | | | PC4 | | | | | | | |  |
| --- | --- | --- | --- | --- | --- | --- | --- | --- | --- | --- | --- | --- | --- | --- | --- | --- | --- | --- | --- | --- |
| Baseline characteristics | Quintile1 | Quintile 2 | | Quintile 3 | | Quintile 4 | | Quintile 5 | | p-trend^†^ | | Quintile1 | Quintile 2 | | Quintile 3 | | Quintile 4 | Quintile 5 | p-trend^†^ | |
| n | 95462 | | 95463 | | 95462 | | 95463 | | 95462 | |  | 95462 | | 95463 | | 95462 | 95463 | 95462 |  | |
| Age ^‡^ | 51.7;9.0 | | 51.8;9.6 | | 51.4;10.1 | | 50.8;10.5 | | 50.2;10.1 | | <0.001 | 49.7;10.6 | | 51.0;9.8 | | 51.4;9.57 | 51.8;9.50 | 51.9;9.9 | <0.001 | |
| Weight ^‡^ | 67.9;13.7 | | 70.0;13.9 | | 70.8;13.7 | | 71.0;13.4 | | 70.9;13.1 | | <0.001 | 69.2;13.5 | | 69.6;13.6 | | 69.8;13.6 | 70.5;13.7 | 71.5;13.7 | <0.001 | |
| Height ^‡^ | 164.0;8.6 | | 165.5;9.0 | | 166.4;9.1 | | 167.0;9.0 | | 166.8;8.6 | | <0.001 | 167.0;8.9 | | 166.1;8.9 | | 165.8;8.9 | 165.7;8.9 | 165.2;9.0 | <0.001 | |
| log (Energy) ^‡^ | 7.7;0.3 | | 7.6;0.3 | | 7.6;0.3 | | 7.6;0.3 | | 7.5;03 | | <0.001 | 7.6;0.3 | | 7.6;0.3 | | 7.6;0.3 | 7.6;0.3 | 7.5;0.3 | <0.001 | |
| Sex |  | |  | |  | |  | |  | |  |  | |  | |  |  |  |  | |
| Male % | 24.9 | | 30.1 | | 31.5 | | 31.8 | | 30.8 | | <0.001 | 29.5 | | 29.1 | | 29.0 | 30.0 | 31.4 |  | |
| Female % | 75.1 | | 69.9 | | 68.5 | | 68.2 | | 69.2 | |  | 70.5 | | 70.9 | | 71.0 | 70.0 | 68.6 | <0.001 | |
| Highest school level |  | |  | |  | |  | |  | |  |  | |  | |  |  |  |  | |
| None-Primary school % | 32.5 | | 30.7 | | 28.2 | | 27.3 | | 31.2 | | <0.001 | 25.2 | | 27.8 | | 29.0 | 31.3 | 36.5 | <0.001 | |
| Technical- Secondary school % | 40.4 | | 42.1 | | 43.8 | | 44.2 | | 43.0 | |  | 46.5 | | 44.3 | | 43.6 | 41.8 | 37.3 |  | |
| Longer education (incl. University deg.) % | 24.4 | | 24.5 | | 24.5 | | 24.2 | | 21.3 | |  | 25.1 | | 25.0 | | 24.3 | 23.3 | 21.1 |  | |
| Physical activity |  | |  | |  | |  | |  | |  |  | |  | |  |  |  |  | |
| Inactive % | 16.4 | | 17.2 | | 16.3 | | 14.0 | | 11.3 | |  | 12.4 | | 14.8 | | 15.6 | 16.3 | 16.1 |  | |
| Moderately inactive % | 39.2 | | 34.1 | | 30.2 | | 25.6 | | 20.9 | | <0.001 | 26.5 | | 31.2 | | 31.6 | 31.0 | 29.6 | <0.001 | |
| Moderately active % | 34.8 | | 35.8 | | 35.3 | | 31.2 | | 27.2 | |  | 29.2 | | 31.4 | | 32.2 | 33.6 | 37.9 |  | |
| Active % | 8.2 | | 9.5 | | 9.5 | | 7.9 | | 6.0 | |  | 8.0 | | 8.0 | | 8.1 | 8.2 | 8.9 |  | |
| Smoking status |  | |  | |  | |  | |  | |  |  | |  | |  |  |  |  | |
| Never smoker % | 52.4 | | 49.1 | | 48.1 | | 48.4 | | 46.3 | |  | 49.4 | | 50.3 | | 49.4 | 48.0 | 47.4 | <0.001 | |
| Former smoker % | 22.8 | | 25.6 | | 27.5 | | 28.3 | | 29.1 | | <0.001 | 26.1 | | 26.3 | | 27.0 | 27.0 | 26.8 |  | |
| Smoker % | 22.1 | | 23.3 | | 22.7 | | 21.6 | | 22.4 | |  | 22.2 | | 21.2 | | 21.5 | 23.1 | 24.3 |  | |

*based on the country-specific FFQ derived intake levels of 23 nutrients, n=477,312

^†^P for trend by Wald test

^‡^mean; SD (standard deviation)
