# Supplementary material for: Nutrient Patterns and Their Food Sources in an International Study Setting: Report from the EPIC Study
Source: PLoS One. 2014 Jun 5;9(6):e98647. doi: 10.1371/journal.pone.0098647 (PMC4047062; doi:10.1371/journal.pone.0098647)
Supplement: Table S3 — Distribution of participants by country and quintiles of nutrient pattern scores in EPIC. (DOCX) [file pone.0098647.s003.docx]

**Table S3. Distribution of participants by country and quintiles of nutrient pattern scores in EPIC*.**

|  | PC1 | | | | | PC2 | | | | |  |
| --- | --- | --- | --- | --- | --- | --- | --- | --- | --- | --- | --- |
| Country | Quintile1 | Quintile 2 | Quintile 3 | Quintile 4 | Quintile 5 | Quintile1 | Quintile 2 | Quintile 3 | Quintile 4 | Quintile 5 | Total |
| France | 5921 (6.2) | 11708 (12.3) | 15297 (16.0) | 18673 (19.6) | 15786 (16.5) | 6543 (6.9) | 10919 (11.4) | 13255 (13.9) | 15774 (16.5) | 20894 (21.9) | 67385 (14.1) |
| Italy | 7616 (8.0) | 10452 (10.9) | 10605 (11.1) | 10079 (10.6) | 5789 (6.1) | 23309 (24.4) | 9748 (10.2) | 5877 (6.2) | 3573 (3.7) | 2034 (2.1) | 44541 (9.3) |
| Spain | 4206 (4.4) | 6274 (6.6) | 8046 (8.4) | 10451 (10.9) | 11025 (11.5) | 15418 (16.2) | 9878 (10.3) | 6738 (7.1) | 4672 (4.9) | 3296 (3.5) | 40002 (8.4) |
| UK General population | 2474 (2.6) | 5554 (5.8) | 7406 (7.8) | 8244 (8.6) | 5829 (6.1) | 1942 (2.0) | 3581 (3.8) | 5254 (5.5) | 7822 (8.2) | 10908 (11.4) | 29507 (6.2) |
| UK Health conscious | 1116 (1.2) | 3535 (3.7) | 6591 (6.9) | 11470 (12.0) | 23176 (24.3) | 4628 (4.8) | 6594 (6.9) | 9426 (9.9) | 12395 (13.0) | 12845 (13.5) | 45888 (9.6) |
| The Netherlands | 3081 (3.2) | 7331 (7.7) | 10231 (10.7) | 10353 (10.8) | 5509 (5.8) | 9618 (10.1) | 10618 (11.1) | 8523 (8.9) | 5571 (5.8) | 2175 (2.3) | 36505 (7.6) |
| Greece | 102 (0.1) | 607 (0.6) | 2040 (2.1) | 4094 (4.3) | 19189 (20.1) | 4948 (5.2) | 7693 (8.1) | 6360 (6.7) | 4460 (4.7) | 2571 (2.7) | 26032 (5.5) |
| Germany | 9219 (9.7) | 15022 (15.7) | 12810 (13.4) | 8388 (8.8) | 3144 (3.3) | 7552 (7.9) | 12073 (12.6) | 12579 (13.2) | 10653 (11.2) | 5726 (6.0) | 48583 (10.2) |
| Sweden | 26111 (27.4) | 11349 (11.9) | 6346 (6.6) | 3555 (3.7) | 1323 (1.4) | 13045 (13.7) | 10857 (11.4) | 9757 (10.2) | 8508 (8.9) | 6517 (6.8) | 48684 (10.2) |
| Denmark | 21439 (22.5) | 13807 (14.5) | 9493 (9.9) | 6650 (7.0) | 3627 (3.8) | 5990 (6.3) | 8525 (8.9) | 10640 (11.1) | 13057 (13.7) | 16804 (17.6) | 55016 (11.5) |
| Norway | 14177 (14.9) | 9824 (10.3) | 6597 (6.9) | 3506 (3.7) | 1065 (1.1) | 2469 (2.6) | 4977 (5.2) | 7053 (7.4) | 8978 (9.4) | 11692 (12.2) | 35169 (7.4) |
| Total | 95462 | 95463 | 95462 | 95463 | 95462 | 95462 | 95463 | 95462 | 95463 | 95462 | 477312 |

*based on the country-specific FFQ derived intake levels of 23 nutrients for 477,312 participants; n (%)

**Table S3.** continued

|  | PC3 | | | | | PC4 | | | | |  |
| --- | --- | --- | --- | --- | --- | --- | --- | --- | --- | --- | --- |
| Country | Quintile1 | Quintile 2 | Quintile 3 | Quintile 4 | Quintile 5 | Quintile1 | Quintile 2 | Quintile 3 | Quintile 4 | Quintile 5 | Total |
| France | 31385 (32.9) | 18315 (19.2) | 10718 (11.2) | 5157 (5.4) | 1810 (1.9) | 10554 (11.1) | 17248 (18.1) | 17002 (17.8) | 13983 (14.6) | 8598 (9.0) | 67385 (14.1) |
| Italy | 19950 (20.9) | 12567 (13.2) | 7277 (7.6) | 3304 (3.5) | 1443 (1.5) | 3482 (3.6) | 8881 (9.3) | 12412 (13.0) | 13081 (13.7) | 6685 (7.0) | 44541 (9.3) |
| Spain | 4863 (5.1) | 5030 (5.3) | 6352 (6.7) | 8240 (8.6) | 15517 (16.3) | 1377 (1.4) | 2806 (2.9) | 4742 (5.0) | 9175 (9.6) | 21902 (22.9) | 40002 (8.4) |
| UK General population | 2712 (2.8) | 4310 (4.5) | 6305 (6.6) | 8389 (8.8) | 7791 (8.2) | 3760 (3.9) | 4097 (4.3) | 4984 (5.2) | 6365 (6.7) | 10301 (10.8) | 29507 (6.2) |
| UK Health conscious | 3369 (3.5) | 5585 (5.9) | 9017 (9.4) | 12828 (13.4) | 15089 (15.8) | 11097 (11.6) | 6717 (7.0) | 6996 (7.3) | 8334 (8.7) | 12744 (13.3) | 45888 (9.6) |
| The Netherlands | 4103 (4.3) | 8802 (9.2) | 12091 (12.7) | 9246 (9.7) | 2263 (2.4) | 10949 (11.5) | 7587 (7.9) | 6564 (6.9) | 6120 (6.4) | 5285 (5.5) | 36505 (7.6) |
| Greece | 10966 (11.5) | 8889 (9.3) | 4504 (4.7) | 1380 (1.4) | 293 (0.3) | 8275 (8.7) | 7820 (8.2) | 5582 (5.) | 3210 (3.4) | 1145 (1.2) | 26032 (5.5) |
| Germany | 7780 (8.1) | 12492 (13.1) | 13318 (14.0) | 9808 (10.3) | 5185 (5.4) | 13816 (14.5) | 13840 (14.5) | 10424 (10.9) | 7114 (7.5) | 3389 (3.6) | 48583 (10.2) |
| Sweden | 1139 (1.2) | 2431 (2.5) | 7241 (7.6) | 18969 (19.9) | 18904 (19.8) | 13581 (14.2) | 10671 (11.2) | 9585 (10.0) | 8695 (9.1) | 6152 (6.4) | 48684 (10.2) |
| Denmark | 8858 (9.3) | 16040 (16.8) | 15659 (16.4) | 9981 (10.5) | 4478 (4.7) | 5198 (5.4) | 8719 (9.1) | 11100 (11.6) | 14075 (14.7) | 15924 (16.7) | 55016 (11.5) |
| Norway | 337 (0.4) | 1002 (1.0) | 2980 (3.1) | 8161 (8.5) | 22689 (23.8) | 13373 (14.0) | 7077 (7.4) | 6071 (6.4) | 5311 (5.6) | 3337 (16.7) | 35169 (7.4) |
| Total | 95462 | 95463 | 95462 | 95463 | 95462 | 95462 | 95463 | 95462 | 95463 | 95462 | 477312 |

*based on the country-specific FFQ derived intake levels of 23 nutrients for 477,312 participants; n (%)
