# Supplementary material for: Nutrient Patterns and Their Food Sources in an International Study Setting: Report from the EPIC Study
Source: PLoS One. 2014 Jun 5;9(6):e98647. doi: 10.1371/journal.pone.0098647 (PMC4047062; doi:10.1371/journal.pone.0098647)
Supplement: Table S4 — Daily mean nutrient intakes in the EPIC Calibration study (EPIC Mean) and per quintiles of PC1 scores and percentage deviation of the quintile mean from the overall EPIC mean. (DOCX) [file pone.0098647.s004.docx]

**Table S4. Daily mean nutrient intakes in the EPIC Calibration study**^†^ **(EPIC Mean) and per quintiles of PC1 scores and percentage deviation of the quintile mean from the overall EPIC mean*.**

| Nutrient | EPIC Mean^†^ | Quintile 1 | | Quintile 2 | | Quintile 3 | | Quintile 4 | | Quintile 5 | |
| --- | --- | --- | --- | --- | --- | --- | --- | --- | --- | --- | --- |
|  |  | Mean^†^ | Deviation | Mean^†^ | Deviation | Mean^†^ |  | Mean^†^ | Deviation | Mean^†^ | Deviation |
| Total proteins, g | 86.7 | 87.3 | 100.8 | 87.4 | 100.8 | 87.1 | 100.5 | 86.4 | 99.7 | 85.1 | 98.2 |
| SFA, g | 30.8 | 34.2 | 111.2 | 31.7 | 103.2 | 30.5 | 99.1 | 29.4 | 95.5 | 28.0 | 91.0 |
| MUFA, g | 32.9 | 34.2 | 104.0 | 32.9 | 100.1 | 32.6 | 99.2 | 32.1 | 97.5 | 32.6 | 99.1 |
| PUFA, g | 13.1 | 13.3 | 102.1 | 13.3 | 101.9 | 13.0 | 99.5 | 12.8 | 97.9 | 12.9 | 98.7 |
| Cholesterol, mg | 322.1 | 356.8 | 110.8 | 331.9 | 103.0 | 322.9 | 100.2 | 312.0 | 96.8 | 287.1 | 89.1 |
| Starch, g | 121.2 | 116.8 | 96.4 | 120.2 | 99.3 | 121.4 | 100.2 | 123.6 | 102.0 | 123.8 | 102.2 |
| Sugar, g | 99.4 | 90.6 | 91.1 | 95.9 | 96.5 | 99.4 | 100.0 | 103.3 | 103.9 | 107.8 | 108.5 |
| Dietary fiber, g | 21.7 | 18.5 | 85.3 | 20.5 | 94.5 | 21.7 | 99.9 | 23.0 | 106.2 | 24.7 | 114.1 |
| Thiamin, mg | 1.3 | 1.2 | 96.9 | 1.2 | 97.9 | 1.3 | 99.5 | 1.3 | 101.8 | 1.3 | 103.9 |
| Riboflavin, mg | 1.7 | 1.7 | 102.4 | 1.7 | 99.8 | 1.7 | 99.0 | 1.7 | 100.2 | 1.7 | 98.5 |
| Vitamin B_6_, mg | 1.8 | 1.7 | 94.5 | 1.7 | 97.6 | 1.8 | 100.0 | 1.8 | 102.5 | 1.9 | 105.5 |
| Folate (Vitamin B_9)_ | 274.8 | 248.5 | 90.4 | 262.2 | 95.4 | 271.6 | 98.8 | 287.5 | 104.6 | 304.2 | 110.7 |
| Vitamin B_12_, µg | 6.4 | 7.7 | 119.0 | 6.7 | 103.5 | 6.2 | 96.9 | 6.1 | 95.0 | 5.5 | 85.6 |
| Vitamin C, mg | 116.8 | 87.3 | 74.7 | 104.4 | 89.4 | 116.1 | 99.4 | 130.4 | 111.7 | 145.7 | 124.8 |
| beta-carotene, µg | 2849.9 | 1941.7 | 68.1 | 2490.5 | 87.4 | 2899.2 | 101.7 | 3164.1 | 111.0 | 3753.8 | 131.7 |
| Retinol, µg | 707.5 | 1007.6 | 142.4 | 736.6 | 104.1 | 667.0 | 94.3 | 609.0 | 86.1 | 517.1 | 73.1 |
| Vitamin E, mg | 11.9 | 11.1 | 93.0 | 11.8 | 99.0 | 11.9 | 99.9 | 12.2 | 102.5 | 12.6 | 105.6 |
| Vitamin D, µg | 3.9 | 4.3 | 110.3 | 3.9 | 99.7 | 3.8 | 99.1 | 3.8 | 97.2 | 3.6 | 93.7 |
| Calcium, mg | 909.8 | 877.5 | 96.4 | 899.5 | 98.9 | 905.6 | 99.5 | 934.7 | 102.7 | 931.8 | 102.4 |
| Phosphorus, mg | 1412.8 | 1393.9 | 98.7 | 1405.5 | 99.5 | 1411.2 | 99.9 | 1429.9 | 101.2 | 1423.3 | 100.7 |
| Iron, mg | 13.0 | 12.5 | 96.2 | 12.9 | 98.8 | 13.0 | 100.1 | 13.3 | 102.2 | 13.4 | 102.8 |
| Potassium, mg | 3556.6 | 3340.6 | 93.9 | 3457.0 | 97.2 | 3549.2 | 99.8 | 3644.4 | 102.5 | 3791.7 | 106.6 |
| Magnesium, mg | 357.0 | 337.5 | 94.6 | 348.8 | 97.7 | 354.4 | 99.3 | 365.9 | 102.5 | 378.1 | 105.9 |

*PC scores calculated on the country-specific FFQ derived intake levels of 23 nutrients, n=477,312

^†^ Mean nutrient intakes in the EPIC Calibration study (n=34,436) adjusted for age, sex, height, weight, total energy intake and centre, weighted for day of the week, and season

^‡^ The adjusted mean values and deviation of the quintile means from the overall EPIC mean are presented graphically in Figure 2
