# Supplementary material for: Nutrient Patterns and Their Food Sources in an International Study Setting: Report from the EPIC Study
Source: PLoS One. 2014 Jun 5;9(6):e98647. doi: 10.1371/journal.pone.0098647 (PMC4047062; doi:10.1371/journal.pone.0098647)
Supplement: Table S5 — Daily mean nutrient intakes in the EPIC Calibration study (EPIC Mean) and per quintiles of PC2 scores and percentage deviation of the quintile mean from the overall EPIC mean. (DOCX) [file pone.0098647.s005.docx]

**Table S5. Daily mean nutrient intakes in the EPIC Calibration study**^†^ **(EPIC Mean) and per quintiles of PC2 scores and percentage deviation of the quintile mean from the overall EPIC mean*.**

| Nutrient | EPIC Mean^†^ | Quintile 1 | | Quintile 2 | | Quintile 3 | | Quintile 4 | | Quintile 5 | |
| --- | --- | --- | --- | --- | --- | --- | --- | --- | --- | --- | --- |
|  |  | Mean^†^ | Deviation | Mean^†^ | Deviation | Mean^†^ |  | Mean^†^ | Deviation | Mean^†^ | Deviation |
| Total proteins, g | 87.3 | 82.9 | 94.9 | 85.5 | 97.9 | 87.4 | 100.1 | 88.9 | 101.9 | 91.8 | 105.2 |
| SFA, g | 30.6 | 31.1 | 101.7 | 31.3 | 102.4 | 31.0 | 101.4 | 30.2 | 98.6 | 29.4 | 96.0 |
| MUFA, g | 32.9 | 32.7 | 99.3 | 33.1 | 100.7 | 33.1 | 100.7 | 32.8 | 99.8 | 32.8 | 99.6 |
| PUFA, g | 13.1 | 12.8 | 97.9 | 13.1 | 100.1 | 13.2 | 100.9 | 13.2 | 100.5 | 13.2 | 100.6 |
| Cholesterol, mg | 324.5 | 305.4 | 94.1 | 318.5 | 98.2 | 330.1 | 101.7 | 331.0 | 102.0 | 337.5 | 104.0 |
| Starch, g | 120.3 | 127.1 | 105.7 | 121.3 | 100.8 | 119.4 | 99.2 | 118.6 | 98.6 | 114.9 | 95.6 |
| Sugar, g | 99.0 | 102.4 | 103.4 | 99.8 | 100.8 | 98.1 | 99.1 | 97.5 | 98.4 | 97.3 | 98.3 |
| Dietary fiber, g | 21.9 | 20.8 | 95.0 | 21.4 | 97.7 | 21.9 | 99.8 | 22.4 | 102.3 | 23.0 | 105.1 |
| Thiamin, mg | 1.3 | 1.2 | 95.2 | 1.2 | 98.0 | 1.3 | 100.8 | 1.3 | 100.8 | 1.3 | 105.2 |
| Riboflavin, mg | 1.7 | 1.6 | 91.2 | 1.7 | 97.0 | 1.7 | 101.1 | 1.8 | 103.1 | 1.8 | 107.6 |
| Vitamin B_6_, mg | 1.8 | 1.7 | 91.9 | 1.8 | 96.9 | 1.8 | 100.4 | 1.9 | 102.7 | 2.0 | 108.1 |
| Folate (Vitamin B_9)_ | 279.0 | 252.7 | 90.5 | 268.8 | 96.3 | 279.3 | 100.1 | 289.5 | 103.7 | 305.1 | 109.3 |
| Vitamin B_12_, µg | 6.6 | 5.5 | 82.6 | 6.2 | 94.1 | 6.9 | 104.7 | 6.9 | 104.6 | 7.5 | 114.0 |
| Vitamin C, mg | 119.7 | 102.5 | 85.6 | 112.1 | 93.7 | 120.5 | 100.7 | 126.3 | 105.5 | 137.1 | 114.5 |
| beta-carotene, µg | 2949.0 | 2427.8 | 82.3 | 2596.5 | 88.0 | 2878.7 | 97.6 | 3126.1 | 106.0 | 3715.8 | 126.0 |
| Retinol, µg | 718.0 | 610.7 | 85.1 | 699.0 | 97.4 | 783.4 | 109.1 | 755.3 | 105.2 | 741.7 | 103.3 |
| Vitamin E, mg | 12.0 | 11.4 | 94.7 | 11.9 | 99.0 | 12.1 | 100.6 | 12.2 | 101.9 | 12.5 | 103.8 |
| Vitamin D, µg | 3.9 | 3.5 | 88.8 | 3.7 | 94.6 | 3.9 | 100.1 | 4.0 | 102.6 | 4.5 | 113.8 |
| Calcium, mg | 920.4 | 852.8 | 92.7 | 896.4 | 97.4 | 918.8 | 99.8 | 949.2 | 103.1 | 984.8 | 107.0 |
| Phosphorus, mg | 1426.9 | 1332.7 | 93.4 | 1394.3 | 97.7 | 1434.5 | 100.5 | 1462.1 | 102.5 | 1511.0 | 105.9 |
| Iron, mg | 13.2 | 12.4 | 94.3 | 12.8 | 97.4 | 13.2 | 100.5 | 13.4 | 101.7 | 14.0 | 106.1 |
| Potassium, mg | 3596.2 | 3345.6 | 93.0 | 3509.5 | 97.6 | 3600.0 | 100.1 | 3678.6 | 102.3 | 3847.3 | 107.0 |
| Magnesium, mg | 360.2 | 340.5 | 94.5 | 352.1 | 97.8 | 358.7 | 99.6 | 368.7 | 102.3 | 381.0 | 105.8 |

*PC scores calculated on the country-specific FFQ derived intake levels of 23 nutrients, n=477,312

^†^ Mean nutrient intakes in the EPIC Calibration study (n=34,436) adjusted for age, sex, height, weight, total energy intake and centre, weighted for day of the week, and season

^‡^ The adjusted mean values and deviation of the quintile means from the overall EPIC mean are presented graphically in Figure 3
