# Supplementary material for: Nutrient Patterns and Their Food Sources in an International Study Setting: Report from the EPIC Study
Source: PLoS One. 2014 Jun 5;9(6):e98647. doi: 10.1371/journal.pone.0098647 (PMC4047062; doi:10.1371/journal.pone.0098647)
Supplement: Table S6 — Daily mean nutrient intakes in the EPIC Calibration study (EPIC Mean) and per quintiles of PC3 scores and percentage deviation of the quintile mean from the overall EPIC mean. (DOCX) [file pone.0098647.s006.docx]

**Table S6. Daily mean nutrient intakes in the EPIC Calibration study**^†^ **(EPIC Mean) and per quintiles of PC3 scores and percentage deviation of the quintile mean from the overall EPIC mean*.**

| Nutrient | EPIC Mean^†^ | Quintile 1 | | Quintile 2 | | Quintile 3 | | Quintile 4 | | Quintile 5 | |
| --- | --- | --- | --- | --- | --- | --- | --- | --- | --- | --- | --- |
|  |  | Mean^†^ | Deviation | Mean^†^ | Deviation | Mean^†^ |  | Mean^†^ | Deviation | Mean^†^ | Deviation |
| Total proteins, g | 86.6 | 86.7 | 100.1 | 86.0 | 99.3 | 86.9 | 100.3 | 86.6 | 100.0 | 86.9 | 100.3 |
| SFA, g | 30.8 | 33.3 | 108.1 | 32.1 | 104.4 | 30.7 | 99.9 | 29.5 | 96.0 | 28.2 | 91.6 |
| MUFA, g | 32.9 | 33.0 | 100.2 | 33.1 | 100.6 | 33.0 | 100.2 | 32.9 | 100.0 | 32.6 | 99.1 |
| PUFA, g | 13.1 | 12.0 | 91.7 | 12.6 | 96.8 | 13.3 | 101.7 | 13.6 | 104.2 | 13.8 | 105.5 |
| Cholesterol, mg | 322.2 | 336.9 | 104.6 | 327.3 | 101.6 | 325.6 | 101.1 | 315.6 | 98.0 | 305.4 | 94.8 |
| Starch, g | 121.2 | 118.0 | 97.4 | 120.0 | 99.0 | 120.9 | 99.8 | 122.6 | 101.2 | 124.4 | 102.7 |
| Sugar, g | 99.5 | 101.0 | 101.5 | 100.1 | 100.6 | 99.0 | 99.5 | 99.0 | 99.5 | 98.3 | 98.8 |
| Dietary fiber, g | 21.7 | 20.8 | 95.9 | 21.4 | 98.7 | 21.4 | 98.6 | 22.2 | 102.1 | 22.7 | 104.7 |
| Thiamin, mg | 1.3 | 1.2 | 99.2 | 1.3 | 99.4 | 1.3 | 99.7 | 1.3 | 100.9 | 1.3 | 100.9 |
| Riboflavin, mg | 1.7 | 1.8 | 104.6 | 1.7 | 101.7 | 1.7 | 99.7 | 1.7 | 98.4 | 1.6 | 95.7 |
| Vitamin B_6_, mg | 1.8 | 1.7 | 97.2 | 1.8 | 98.3 | 1.8 | 99.5 | 1.8 | 101.5 | 1.8 | 103.5 |
| Folate (Vitamin B_9)_ | 274.8 | 272.2 | 99.1 | 273.9 | 99.6 | 271.4 | 98.7 | 276.7 | 100.7 | 280.1 | 101.9 |
| Vitamin B_12_, µg | 6.4 | 6.7 | 104.7 | 6.3 | 98.5 | 6.4 | 100.0 | 6.3 | 98.0 | 6.4 | 98.8 |
| Vitamin C, mg | 116.8 | 113.1 | 96.8 | 114.8 | 98.3 | 114.5 | 98.0 | 117.5 | 100.6 | 124.2 | 106.3 |
| beta-carotene, µg | 2855.6 | 2770.4 | 97.0 | 2779.1 | 97.3 | 2835.6 | 99.3 | 2929.3 | 102.6 | 2963.6 | 103.8 |
| Retinol, µg | 706.3 | 860.5 | 121.8 | 763.0 | 108.0 | 698.3 | 98.9 | 642.8 | 91.0 | 567.1 | 80.3 |
| Vitamin E, mg | 11.9 | 10.9 | 91.4 | 11.4 | 95.9 | 12.0 | 100.8 | 12.5 | 104.9 | 12.7 | 106.9 |
| Vitamin D, µg | 3.8 | 3.2 | 83.7 | 3.3 | 86.2 | 3.7 | 96.6 | 4.2 | 109.3 | 4.8 | 124.3 |
| Calcium, mg | 910.5 | 953.7 | 104.7 | 920.6 | 101.1 | 911.0 | 100.0 | 896.4 | 98.4 | 871.1 | 95.7 |
| Phosphorus, mg | 1413.1 | 1425.5 | 100.9 | 1408.0 | 99.6 | 1420.9 | 100.6 | 1407.2 | 99.6 | 1403.9 | 99.4 |
| Iron, mg | 13.0 | 12.8 | 97.9 | 12.9 | 99.0 | 13.0 | 99.8 | 13.1 | 100.6 | 13.4 | 102.7 |
| Potassium, mg | 3556.8 | 3510.6 | 98.7 | 3517.3 | 98.9 | 3543.3 | 99.6 | 3583.6 | 100.8 | 3629.3 | 102.0 |
| Magnesium, mg | 356.9 | 348.9 | 97.8 | 352.7 | 98.8 | 356.4 | 99.8 | 359.3 | 100.7 | 367.4 | 102.9 |

*PC scores calculated on the country-specific FFQ derived intake levels of 23 nutrients, n=477,312

^†^ Mean nutrient intakes in the EPIC Calibration study (n=34,436) adjusted for age, sex, height, weight, total energy intake and centre, weighted for day of the week, and season

^‡^ The adjusted mean values and deviation of the quintile means from the overall EPIC mean are presented graphically in Figure 4
