# Supplementary material for: Nutrient Patterns and Their Food Sources in an International Study Setting: Report from the EPIC Study
Source: PLoS One. 2014 Jun 5;9(6):e98647. doi: 10.1371/journal.pone.0098647 (PMC4047062; doi:10.1371/journal.pone.0098647)
Supplement: Table S7 — Daily mean nutrient intakes in the EPIC Calibration study (EPIC Mean) and per quintiles of PC4 scores and percentage deviation of the quintile mean from the overall EPIC mean. (DOCX) [file pone.0098647.s007.docx]

**Table S7. Daily mean nutrient intakes in the EPIC Calibration study**^†^ **(EPIC Mean) and per quintiles of PC4 scores and percentage deviation of the quintile mean from the overall EPIC mean*.**

| Nutrient | EPIC Mean^†^ | Quintile 1 | | Quintile 2 | | Quintile 3 | | Quintile 4 | | Quintile 5 | |
| --- | --- | --- | --- | --- | --- | --- | --- | --- | --- | --- | --- |
|  |  | Mean^†^ | Deviation | Mean^†^ | Deviation | Mean^†^ |  | Mean^†^ | Deviation | Mean^†^ | Deviation |
| Total proteins, g | 86.4 | 82.7 | 95.7 | 84.8 | 98.1 | 87.0 | 100.6 | 87.7 | 101.5 | 89.9 | 104.1 |
| SFA, g | 30.8 | 31.1 | 101.2 | 31.0 | 100.7 | 31.0 | 100.8 | 30.8 | 100.0 | 29.9 | 97.3 |
| MUFA, g | 32.9 | 33.3 | 101.3 | 33.2 | 100.9 | 33.0 | 100.4 | 32.8 | 99.6 | 32.2 | 97.9 |
| PUFA, g | 13.1 | 14.6 | 110.9 | 13.5 | 102.5 | 13.0 | 99.2 | 12.7 | 96.4 | 12.0 | 91.1 |
| Cholesterol, mg | 321.5 | 313.4 | 97.5 | 316.3 | 98.4 | 327.6 | 101.9 | 324.4 | 100.9 | 325.7 | 101.3 |
| Starch, g | 121.5 | 125.6 | 103.4 | 124.3 | 102.3 | 121.2 | 99.8 | 119.6 | 98.5 | 116.6 | 96.0 |
| Sugar, g | 99.4 | 99.5 | 100.1 | 98.6 | 99.2 | 97.6 | 98.1 | 99.7 | 100.3 | 101.7 | 102.3 |
| Dietary fiber, g | 21.7 | 22.3 | 102.4 | 21.9 | 100.9 | 21.6 | 99.4 | 21.6 | 99.3 | 21.3 | 98.0 |
| Thiamin, mg | 1.3 | 1.2 | 97.6 | 1.2 | 98.0 | 1.3 | 100.6 | 1.3 | 101.3 | 1.3 | 102.5 |
| Riboflavin, mg | 1.7 | 1.5 | 90.4 | 1.6 | 94.9 | 1.7 | 100.0 | 1.7 | 104.1 | 1.8 | 110.5 |
| Vitamin B_6_, mg | 1.8 | 1.7 | 97.0 | 1.8 | 98.3 | 1.8 | 99.8 | 1.8 | 101.4 | 1.8 | 103.4 |
| Folate (Vitamin B_9)_ | 274.7 | 271.2 | 98.7 | 271.2 | 98.7 | 275.0 | 100.1 | 276.0 | 100.5 | 280.2 | 102.0 |
| Vitamin B_12_, µg | 6.4 | 5.8 | 91.3 | 6.1 | 94.6 | 6.5 | 101.2 | 6.7 | 105.1 | 6.9 | 107.8 |
| Vitamin C, mg | 116.7 | 111.9 | 95.9 | 113.6 | 97.3 | 116.3 | 99.6 | 119.3 | 102.2 | 122.5 | 105.0 |
| beta-carotene, µg | 2877.7 | 3272.8 | 113.7 | 2931.9 | 101.9 | 2868.8 | 99.7 | 2726.5 | 94.7 | 2588.7 | 90.0 |
| Retinol, µg | 707.6 | 748.4 | 105.8 | 713.1 | 100.8 | 715.5 | 101.1 | 689.8 | 97.5 | 671.4 | 94.9 |
| Vitamin E, mg | 12.0 | 13.1 | 109.3 | 12.2 | 102.0 | 11.9 | 99.1 | 11.6 | 96.9 | 11.1 | 92.8 |
| Vitamin D, µg | 3.9 | 3.9 | 101.4 | 3.8 | 97.4 | 3.8 | 98.3 | 3.9 | 101.6 | 3.9 | 101.2 |
| Calcium, mg | 903.3 | 792.6 | 87.8 | 857.3 | 94.9 | 900.2 | 99.7 | 950.9 | 105.3 | 1015.4 | 112.4 |
| Phosphorus, mg | 1407.1 | 1310.4 | 93.1 | 1366.5 | 97.1 | 1411.8 | 100.3 | 1441.8 | 102.5 | 1504.9 | 107.0 |
| Iron, mg | 13.0 | 12.9 | 98.9 | 12.9 | 99.3 | 13.0 | 99.7 | 13.1 | 100.6 | 13.2 | 101.6 |
| Potassium, mg | 3549.3 | 3414.7 | 96.2 | 3472.4 | 97.8 | 3540.2 | 99.7 | 3616.8 | 101.9 | 3702.4 | 104.3 |
| Magnesium, mg | 356.3 | 343.4 | 96.4 | 349.1 | 98.0 | 356.0 | 99.9 | 361.5 | 101.5 | 371.3 | 104.2 |

*PC scores calculated on the country-specific FFQ derived intake levels of 23 nutrients, n=477,312

^†^ Mean nutrient intakes in the EPIC Calibration study (n=34,436) adjusted for age, sex, height, weight, total energy intake and centre, weighted for day of the week, and season

^‡^ The adjusted mean values and deviation of the quintile means from the overall EPIC mean are presented graphically in Figure 5
