# Supplementary material for: Nutrient Patterns and Their Food Sources in an International Study Setting: Report from the EPIC Study
Source: PLoS One. 2014 Jun 5;9(6):e98647. doi: 10.1371/journal.pone.0098647 (PMC4047062; doi:10.1371/journal.pone.0098647)
Supplement: Table S8 — Daily means of food/food group intakes in the EPIC Calibration study (EPIC Mean) and per quintiles of PC1 scores and percentage deviation of the quintile mean from the overall EPIC mean. (DOCX) [file pone.0098647.s008.docx]

**Table S8. Daily means of food/food group intakes in the EPIC Calibration study**^†^ **(EPIC Mean) and per quintiles of PC1 scores and percentage deviation of the quintile mean from the overall EPIC mean*.**

| Food/Food group | | EPIC Mean^†^ | | Quintile 1 | | | | Quintile 2 | | | | Quintile 3 | | | | Quintile 4 | | | | Quintile 5 | | | |
| --- | --- | --- | --- | --- | --- | --- | --- | --- | --- | --- | --- | --- | --- | --- | --- | --- | --- | --- | --- | --- | --- | --- | --- |
|  | |  | | Mean^†^ | | Deviation | | Mean^†^ | | Deviation | | Mean^†^ | | Deviation | | Mean^†^ | | Deviation | | Mean^†^ | | Deviation | |
| Alcohol, g | 15.5 | | 15.9 | | 102.6 | | 16.0 | | 102.8 | | 15.9 | | 102.6 | | 15.3 | | 98.6 | | 14.5 | | 93.4 | |  |
| Potatoes & Other tubers, g | 73.3 | | 75.5 | | 103.0 | | 73.2 | | 99.9 | | 74.6 | | 101.8 | | 72.6 | | 99.0 | | 70.6 | | 96.3 | |  |
| Vegetables, g | 178.2 | | 140.1 | | 78.6 | | 161.9 | | 90.8 | | 175.8 | | 98.7 | | 191.8 | | 107.7 | | 221.4 | | 124.2 | |  |
| Legumes, g | 14.6 | | 13.7 | | 93.7 | | 14.6 | | 100.2 | | 15.0 | | 102.5 | | 14.4 | | 98.4 | | 15.4 | | 105.3 | |  |
| Fruits, g | 253.6 | | 181.3 | | 71.5 | | 223.7 | | 88.2 | | 252.8 | | 99.7 | | 284.9 | | 112.4 | | 325.3 | | 128.3 | |  |
| Other Dairy Products, g | 111.3 | | 97.1 | | 87.3 | | 109.0 | | 97.9 | | 112.9 | | 101.5 | | 118.5 | | 106.5 | | 118.8 | | 106.8 | |  |
| Milk, g | 174.6 | | 201.5 | | 115.4 | | 176.8 | | 101.3 | | 168.0 | | 96.2 | | 166.4 | | 95.3 | | 160.3 | | 91.8 | |  |
| Cereals & Cereal products, g | 209.0 | | 196.4 | | 94.0 | | 205.0 | | 98.1 | | 209.1 | | 100.0 | | 216.8 | | 103.8 | | 217.7 | | 104.2 | |  |
| Fresh Meat, g | 72.4 | | 77.8 | | 107.4 | | 75.8 | | 104.7 | | 73.6 | | 101.7 | | 68.5 | | 94.6 | | 66.3 | | 91.6 | |  |
| Processed Meat, g | 38.4 | | 48.5 | | 126.4 | | 40.8 | | 106.3 | | 38.4 | | 100.0 | | 33.5 | | 87.4 | | 30.7 | | 79.9 | |  |
| Fish & Shellfish, g | 40.6 | | 38.9 | | 95.7 | | 39.9 | | 98.2 | | 40.6 | | 99.8 | | 42.3 | | 104.0 | | 41.6 | | 102.3 | |  |
| Eggs, g | 15.7 | | 17.8 | | 113.5 | | 16.0 | | 102.2 | | 16.0 | | 101.9 | | 15.4 | | 98.5 | | 13.2 | | 84.0 | |  |
| Vegetable oils, g | 13.1 | | 11.3 | | 85.9 | | 12.5 | | 95.2 | | 13.3 | | 101.8 | | 13.7 | | 104.2 | | 14.8 | | 112.9 | |  |
| Butter, g | 4.5 | | 6.2 | | 137.2 | | 5.3 | | 117.2 | | 4.4 | | 96.8 | | 3.9 | | 84.8 | | 2.9 | | 64.0 | |  |
| Sugar & Confectionary, g | 27.1 | | 29.3 | | 108.2 | | 27.2 | | 100.2 | | 26.8 | | 98.9 | | 26.3 | | 97.1 | | 25.9 | | 95.6 | |  |
| Cakes, g | 45.8 | | 46.0 | | 100.4 | | 47.4 | | 103.4 | | 46.7 | | 101.8 | | 45.1 | | 98.5 | | 44.0 | | 95.9 | |  |
| Fruit & vegetable juices, g | 56.1 | | 30.7 | | 54.7 | | 47.8 | | 85.3 | | 57.5 | | 102.5 | | 70.7 | | 126.0 | | 73.7 | | 131.5 | |  |
| Carbon. Soft drinks Syrups, g | 67.9 | | 71.5 | | 105.3 | | 66.8 | | 98.5 | | 68.2 | | 100.5 | | 66.1 | | 97.4 | | 66.8 | | 98.4 | |  |
| Margarines, g | 561.2 | | 572.4 | | 102.0 | | 539.1 | | 96.1 | | 549.5 | | 97.9 | | 562.7 | | 100.3 | | 582.2 | | 103.7 | |  |
| Coffee, g | 345.9 | | 402.2 | | 116.3 | | 348.7 | | 100.8 | | 336.7 | | 97.4 | | 325.3 | | 94.0 | | 316.6 | | 91.5 | |  |
| Tea, g | 166.0 | | 139.3 | | 83.9 | | 156.4 | | 94.2 | | 167.6 | | 101.0 | | 180.4 | | 108.6 | | 186.5 | | 112.3 | |  |
| Sauces, g | 29.0 | | 30.5 | | 105.1 | | 29.1 | | 100.2 | | 28.2 | | 97.1 | | 28.0 | | 96.6 | | 29.3 | | 101.0 | |  |
| Soy products, g | 5.4 | | 4.2 | | 76.4 | | 4.3 | | 78.5 | | 4.4 | | 81.6 | | 5.3 | | 98.4 | | 9.0 | | 165.1 | |  |

*PC scores calculated on the country-specific FFQ derived intake levels of 23 nutrients, n=477,312

^†^ Mean nutrient intakes in the EPIC Calibration study (n=34,436) adjusted for age, sex, height, weight, total energy intake and centre, weighted for day of the week, and season

^‡^ The adjusted mean values and deviation of the quintile means from the overall EPIC mean are presented graphically in Figure 2
