# Supplementary material for: Nutrient Patterns and Their Food Sources in an International Study Setting: Report from the EPIC Study
Source: PLoS One. 2014 Jun 5;9(6):e98647. doi: 10.1371/journal.pone.0098647 (PMC4047062; doi:10.1371/journal.pone.0098647)
Supplement: Table S9 — Daily means of food/food group intakes in the EPIC Calibration study (EPIC Mean) and per quintiles of PC2 scores and percentage deviation of the quintile mean from the overall EPIC mean. (DOCX) [file pone.0098647.s009.docx]

**Table S9. Daily means of food/food group intakes in the EPIC Calibration study**^†^ **(EPIC Mean) and per quintiles of PC2 scores and percentage deviation of the quintile mean from the overall EPIC mean*.**

| Food/Food group | | EPIC Mean^†^ | | Quintile 1 | | | | Quintile 2 | | | | Quintile 3 | | | | Quintile 4 | | | | Quintile 5 | | | |
| --- | --- | --- | --- | --- | --- | --- | --- | --- | --- | --- | --- | --- | --- | --- | --- | --- | --- | --- | --- | --- | --- | --- | --- |
|  | |  | | Mean^†^ | | Deviation | | Mean^†^ | | Deviation | | Mean^†^ | | Deviation | | Mean^†^ | | Deviation | | Mean^†^ | | Deviation | |
| Alcohol, g | 16.0 | | 13.0 | | 81.1 | | 14.8 | | 92.4 | | 16.0 | | 99.7 | | 17.4 | | 108.7 | | 18.9 | | 118.1 | |  |
| Potatoes & Other tubers, g | 73.2 | | 72.2 | | 98.6 | | 75.8 | | 103.5 | | 75.7 | | 103.5 | | 72.3 | | 98.8 | | 70.0 | | 95.7 | |  |
| Vegetables, g | 183.7 | | 151.9 | | 82.7 | | 166.8 | | 90.8 | | 181.7 | | 98.9 | | 197.5 | | 107.5 | | 220.6 | | 120.1 | |  |
| Legumes, g | 14.6 | | 15.3 | | 104.6 | | 13.7 | | 94.3 | | 14.4 | | 99.0 | | 14.1 | | 96.6 | | 15.4 | | 105.5 | |  |
| Fruits, g | 258.4 | | 231.5 | | 89.6 | | 247.8 | | 95.9 | | 258.4 | | 100.0 | | 267.9 | | 103.7 | | 286.2 | | 110.8 | |  |
| Other Dairy Products, g | 113.3 | | 100.3 | | 88.5 | | 109.7 | | 96.8 | | 113.8 | | 100.5 | | 115.1 | | 101.6 | | 127.7 | | 112.7 | |  |
| Milk, g | 176.6 | | 161.6 | | 91.5 | | 172.7 | | 97.8 | | 175.5 | | 99.4 | | 185.1 | | 104.8 | | 187.9 | | 106.4 | |  |
| Cereals & Cereal products, g | 207.7 | | 219.7 | | 105.8 | | 206.8 | | 99.6 | | 204.9 | | 98.7 | | 207.1 | | 99.7 | | 199.9 | | 96.2 | |  |
| Fresh Meat, g | 73.1 | | 68.5 | | 93.7 | | 70.9 | | 97.0 | | 72.9 | | 99.7 | | 74.9 | | 102.4 | | 78.3 | | 107.2 | |  |
| Processed Meat, g | 38.5 | | 37.0 | | 96.1 | | 38.0 | | 98.7 | | 39.7 | | 103.3 | | 38.4 | | 99.9 | | 39.2 | | 101.9 | |  |
| Fish & Shellfish, g | 41.8 | | 34.6 | | 82.7 | | 39.0 | | 93.2 | | 41.8 | | 100.1 | | 43.9 | | 105.0 | | 49.7 | | 119.0 | |  |
| Eggs, g | 15.9 | | 14.0 | | 87.6 | | 15.0 | | 94.3 | | 16.4 | | 102.7 | | 16.7 | | 105.0 | | 17.6 | | 110.4 | |  |
| Vegetable oils, g | 13.2 | | 12.5 | | 94.1 | | 12.8 | | 96.7 | | 13.4 | | 101.0 | | 13.5 | | 102.3 | | 14.0 | | 105.9 | |  |
| Butter, g | 4.4 | | 5.5 | | 125.8 | | 4.7 | | 108.7 | | 4.7 | | 107.7 | | 3.7 | | 85.4 | | 3.2 | | 72.5 | |  |
| Sugar & Confectionary, g | 26.0 | | 33.5 | | 129.0 | | 28.8 | | 111.0 | | 25.3 | | 97.2 | | 23.3 | | 89.5 | | 19.0 | | 73.2 | |  |
| Cakes, g | 45.0 | | 50.2 | | 111.6 | | 48.0 | | 106.7 | | 44.4 | | 98.7 | | 42.5 | | 94.6 | | 39.8 | | 88.4 | |  |
| Fruit & vegetable juices, g | 57.5 | | 48.7 | | 84.7 | | 55.0 | | 95.6 | | 61.1 | | 106.3 | | 59.2 | | 103.0 | | 63.6 | | 110.5 | |  |
| Carbon. Soft drinks Syrups, g | 64.3 | | 92.2 | | 143.4 | | 66.4 | | 103.2 | | 59.2 | | 92.2 | | 54.5 | | 84.7 | | 49.2 | | 76.5 | |  |
| Margarines, g | 562.7 | | 549.7 | | 97.7 | | 566.6 | | 100.7 | | 561.4 | | 99.8 | | 564.8 | | 100.4 | | 571.1 | | 101.5 | |  |
| Coffee, g | 344.8 | | 343.4 | | 99.6 | | 357.4 | | 103.6 | | 349.2 | | 101.3 | | 338.8 | | 98.2 | | 335.5 | | 97.3 | |  |
| Tea, g | 168.1 | | 155.7 | | 92.6 | | 165.2 | | 98.2 | | 166.0 | | 98.7 | | 173.4 | | 103.1 | | 180.4 | | 107.3 | |  |
| Sauces, g | 29.0 | | 29.1 | | 100.3 | | 29.1 | | 100.3 | | 29.2 | | 100.9 | | 29.2 | | 100.8 | | 28.3 | | 97.6 | |  |
| Soy products, g | 5.3 | | 6.3 | | 118.0 | | 5.6 | | 105.9 | | 5.4 | | 102.7 | | 4.6 | | 86.9 | | 4.6 | | 86.5 | |  |

*PC scores calculated on the country-specific FFQ derived intake levels of 23 nutrients, n=477,312

^†^ Mean nutrient intakes in the EPIC Calibration study (n=34,436) adjusted for age, sex, height, weight, total energy intake and centre, weighted for day of the week, and season

^‡^ The adjusted mean values and deviation of the quintile means from the overall EPIC mean are presented graphically in Figure 3
