# Supplementary material for: Nutrient Patterns and Their Food Sources in an International Study Setting: Report from the EPIC Study
Source: PLoS One. 2014 Jun 5;9(6):e98647. doi: 10.1371/journal.pone.0098647 (PMC4047062; doi:10.1371/journal.pone.0098647)
Supplement: Table S10 — Daily means of food/food group intakes in the EPIC Calibration study (EPIC Mean) and per quintiles of PC3 scores and percentage deviation of the quintile mean from the overall EPIC mean. (DOCX) [file pone.0098647.s010.docx]

**Table S10. Daily means of food/food group intakes in the EPIC Calibration study**^†^ **(EPIC Mean) and per quintiles of PC3 scores and percentage deviation of the quintile mean from the overall EPIC mean*.**

| Food/Food group | | EPIC Mean^†^ | | Quintile 1 | | | | Quintile 2 | | | | Quintile 3 | | | | Quintile 4 | | | | Quintile 5 | | | |
| --- | --- | --- | --- | --- | --- | --- | --- | --- | --- | --- | --- | --- | --- | --- | --- | --- | --- | --- | --- | --- | --- | --- | --- |
|  | |  | | Mean^†^ | | Deviation | | Mean^†^ | | Deviation | | Mean^†^ | | Deviation | | Mean^†^ | | Deviation | | Mean^†^ | | Deviation | |
| Alcohol, g | 15.5 | | 14.4 | | 93.0 | | 14.8 | | 95.1 | | 15.4 | | 99.4 | | 15.9 | | 102.7 | | 17.0 | | 109.8 | |  |
| Potatoes & Other tubers, g | 73.2 | | 72.7 | | 99.3 | | 71.4 | | 97.5 | | 71.2 | | 97.3 | | 75.3 | | 102.8 | | 75.5 | | 103.1 | |  |
| Vegetables, g | 178.5 | | 166.0 | | 93.0 | | 173.4 | | 97.2 | | 179.6 | | 100.7 | | 183.9 | | 103.1 | | 189.3 | | 106.1 | |  |
| Legumes, g | 14.6 | | 14.8 | | 101.4 | | 14.3 | | 97.8 | | 14.6 | | 99.7 | | 14.6 | | 100.3 | | 14.7 | | 100.8 | |  |
| Fruits, g | 253.9 | | 245.4 | | 96.7 | | 252.4 | | 99.4 | | 247.8 | | 97.6 | | 256.9 | | 101.2 | | 266.9 | | 105.1 | |  |
| Other Dairy Products, g | 111.7 | | 112.4 | | 100.7 | | 112.9 | | 101.1 | | 112.7 | | 100.9 | | 115.7 | | 103.6 | | 104.7 | | 93.8 | |  |
| Milk, g | 174.6 | | 207.7 | | 119.0 | | 188.1 | | 107.7 | | 171.7 | | 98.3 | | 160.9 | | 92.1 | | 144.7 | | 82.9 | |  |
| Cereals & Cereal products, g | 209.0 | | 200.7 | | 96.0 | | 207.0 | | 99.0 | | 208.2 | | 99.6 | | 211.2 | | 101.1 | | 217.9 | | 104.3 | |  |
| Fresh Meat, g | 72.4 | | 74.1 | | 102.4 | | 72.4 | | 100.1 | | 72.7 | | 100.4 | | 71.7 | | 99.1 | | 70.9 | | 98.0 | |  |
| Processed Meat, g | 38.3 | | 41.9 | | 109.2 | | 39.7 | | 103.6 | | 39.1 | | 101.9 | | 36.7 | | 95.7 | | 34.4 | | 89.6 | |  |
| Fish & Shellfish, g | 40.5 | | 32.1 | | 79.2 | | 35.3 | | 87.2 | | 41.4 | | 102.2 | | 43.3 | | 107.0 | | 50.4 | | 124.5 | |  |
| Eggs, g | 15.7 | | 15.7 | | 100.2 | | 15.7 | | 100.0 | | 16.3 | | 103.9 | | 15.6 | | 99.4 | | 15.1 | | 96.5 | |  |
| Vegetable oils, g | 13.1 | | 12.3 | | 93.7 | | 12.9 | | 98.1 | | 13.1 | | 100.1 | | 13.6 | | 103.5 | | 13.7 | | 104.5 | |  |
| Butter, g | 4.5 | | 7.1 | | 156.6 | | 6.1 | | 134.6 | | 4.0 | | 88.5 | | 3.1 | | 67.7 | | 2.4 | | 52.6 | |  |
| Sugar & Confectionary, g | 27.1 | | 29.3 | | 108.2 | | 27.1 | | 100.3 | | 27.4 | | 101.1 | | 25.5 | | 94.2 | | 26.0 | | 96.2 | |  |
| Cakes, g | 45.9 | | 45.1 | | 98.3 | | 45.9 | | 100.0 | | 46.0 | | 100.3 | | 46.6 | | 101.6 | | 45.7 | | 99.8 | |  |
| Fruit & vegetable juices, g | 56.2 | | 55.2 | | 98.2 | | 54.8 | | 97.4 | | 53.3 | | 94.8 | | 59.0 | | 105.0 | | 58.8 | | 104.6 | |  |
| Carbon. Soft drinks Syrups, g | 68.2 | | 72.2 | | 105.9 | | 73.3 | | 107.4 | | 70.5 | | 103.4 | | 67.1 | | 98.4 | | 57.8 | | 84.8 | |  |
| Margarines, g | 561.4 | | 558.1 | | 99.4 | | 556.5 | | 99.1 | | 568.9 | | 101.3 | | 561.8 | | 100.1 | | 561.6 | | 100.0 | |  |
| Coffee, g | 346.0 | | 355.4 | | 102.7 | | 350.8 | | 101.4 | | 357.6 | | 103.4 | | 338.0 | | 97.7 | | 328.3 | | 94.9 | |  |
| Tea, g | 166.3 | | 159.0 | | 95.6 | | 161.0 | | 96.8 | | 164.9 | | 99.2 | | 174.3 | | 104.8 | | 172.4 | | 103.6 | |  |
| Sauces, g | 29.0 | | 26.9 | | 92.5 | | 28.4 | | 97.9 | | 29.9 | | 102.9 | | 30.0 | | 103.3 | | 30.0 | | 103.2 | |  |
| Soy products, g | 5.4 | | 4.5 | | 83.6 | | 4.7 | | 87.0 | | 4.8 | | 88.9 | | 5.7 | | 106.0 | | 7.2 | | 134.6 | |  |

*PC scores calculated on the country-specific FFQ derived intake levels of 23 nutrients, n=477,312

^†^ Mean nutrient intakes in the EPIC Calibration study (n=34,436) adjusted for age, sex, height, weight, total energy intake and centre, weighted for day of the week, and season

^‡^ The adjusted mean values and deviation of the quintile means from the overall EPIC mean are presented graphically in Figure 4
