# Supplementary material for: Nutrient Patterns and Their Food Sources in an International Study Setting: Report from the EPIC Study
Source: PLoS One. 2014 Jun 5;9(6):e98647. doi: 10.1371/journal.pone.0098647 (PMC4047062; doi:10.1371/journal.pone.0098647)
Supplement: Table S11 — Daily means of food/food group intakes in the EPIC calibration study (EPIC Mean) and per quintiles of PC4 scores and percentage deviation of the quintile mean from the overall EPIC mean. (DOCX) [file pone.0098647.s011.docx]

**Table S11. Daily means of food/food group intakes in the EPIC Calibration study**^†^ **(EPIC Mean) and per quintiles of PC4 scores and percentage deviation of the quintile mean from the overall EPIC mean*.**

| Food/Food group | EPIC Mean^†^ | Quintile 1 | | Quintile 2 | | Quintile 3 | | Quintile 4 | | Quintile 5 | |
| --- | --- | --- | --- | --- | --- | --- | --- | --- | --- | --- | --- |
|  |  | Mean^†^ | Deviation | Mean^†^ | Deviation | Mean^†^ | Deviation | Mean^†^ | Deviation | Mean^†^ | Deviation |
| Alcohol, g | 15.4 | 12.2 | 79.6 | 14.1 | 91.7 | 15.8 | 102.7 | 16.3 | 106.0 | 18.4 | 120.0 |
| Potatoes & Other tubers, g | 73.6 | 77.8 | 105.7 | 76.8 | 104.3 | 73.4 | 99.7 | 73.9 | 100.4 | 66.2 | 89.9 |
| Vegetables, g | 179.0 | 185.7 | 103.8 | 183.0 | 102.2 | 179.8 | 100.5 | 175.7 | 98.2 | 170.7 | 95.4 |
| Legumes, g | 14.6 | 14.8 | 101.2 | 14.4 | 98.5 | 14.2 | 97.6 | 14.3 | 98.2 | 15.3 | 104.6 |
| Fruits, g | 253.8 | 248.0 | 97.7 | 251.7 | 99.2 | 250.6 | 98.7 | 259.8 | 102.4 | 259.0 | 102.0 |
| Other Dairy Products, g | 110.5 | 95.2 | 86.2 | 104.8 | 94.9 | 110.0 | 99.6 | 118.7 | 107.5 | 123.6 | 111.9 |
| Milk, g | 170.2 | 107.2 | 63.0 | 136.3 | 80.1 | 167.5 | 98.5 | 195.2 | 114.7 | 244.6 | 143.7 |
| Cereals & Cereal products, g | 209.5 | 215.2 | 102.7 | 213.3 | 101.8 | 209.2 | 99.9 | 205.6 | 98.2 | 204.1 | 97.4 |
| Fresh Meat, g | 72.2 | 68.9 | 95.5 | 71.9 | 99.6 | 73.4 | 101.7 | 73.5 | 101.7 | 73.3 | 101.5 |
| Processed Meat, g | 38.3 | 38.7 | 101.1 | 38.6 | 100.7 | 38.4 | 100.3 | 38.7 | 101.0 | 37.1 | 96.9 |
| Fish & Shellfish, g | 40.4 | 37.5 | 92.6 | 37.5 | 92.8 | 41.4 | 102.2 | 41.1 | 101.6 | 44.8 | 110.7 |
| Eggs, g | 15.6 | 15.2 | 97.3 | 15.2 | 97.5 | 16.0 | 102.5 | 15.5 | 98.9 | 16.2 | 103.8 |
| Vegetable oils, g | 13.1 | 13.1 | 99.6 | 13.4 | 102.0 | 13.2 | 100.2 | 13.0 | 98.7 | 13.1 | 99.5 |
| Butter, g | 4.6 | 5.0 | 109.3 | 4.7 | 103.5 | 4.6 | 101.7 | 4.3 | 93.5 | 4.2 | 92.0 |
| Sugar & Confectionary, g | 27.3 | 30.4 | 111.5 | 28.7 | 105.3 | 26.0 | 95.3 | 26.0 | 95.3 | 25.2 | 92.6 |
| Cakes, g | 46.2 | 51.8 | 112.2 | 48.0 | 104.0 | 46.5 | 100.6 | 43.8 | 94.9 | 40.8 | 88.3 |
| Fruit & vegetable juices, g | 55.8 | 47.4 | 84.9 | 53.4 | 95.6 | 55.7 | 99.8 | 60.6 | 108.5 | 62.0 | 111.1 |
| Carbon. Soft drinks Syrups, g | 68.3 | 78.0 | 114.1 | 72.4 | 105.9 | 63.8 | 93.4 | 62.8 | 92.0 | 64.7 | 94.6 |
| Margarines, g | 561.3 | 568.8 | 101.3 | 554.8 | 98.8 | 558.4 | 99.5 | 561.6 | 100.1 | 562.8 | 100.3 |
| Coffee, g | 345.2 | 339.8 | 98.4 | 343.5 | 99.5 | 349.5 | 101.2 | 342.9 | 99.3 | 350.5 | 101.5 |
| Tea, g | 166.4 | 170.3 | 102.3 | 166.1 | 99.8 | 162.3 | 97.6 | 168.7 | 101.4 | 164.5 | 98.9 |
| Sauces, g | 29.1 | 29.6 | 101.9 | 30.4 | 104.5 | 28.9 | 99.3 | 28.3 | 97.4 | 28.2 | 96.9 |
| Soy products, g | 5.5 | 7.7 | 140.2 | 5.0 | 91.1 | 5.1 | 92.4 | 4.9 | 89.1 | 4.8 | 87.3 |

*PC scores calculated on the country-specific FFQ derived intake levels of 23 nutrients, n=477,312

^†^ Mean nutrient intakes in the EPIC Calibration study (n=34,436) adjusted for age, sex, height, weight, total energy intake and centre, weighted for day of the week, and season

^‡^ The adjusted mean values and deviation of the quintile means from the overall EPIC mean are presented graphically in Figure 5
